# Genetic support of the causal association between gut microbiota and peripheral artery disease: a bidirectional Mendelian randomization study

**DOI:** 10.18632/aging.205417

**Published:** 2024-01-09

**Authors:** Hongshuo Shi, Xin Yuan, Fangfang Wu, Xiaoyu Li, Weijing Fan, Xiao Yang, Guobin Liu

**Affiliations:** 1 Department of Peripheral Vascular Surgery, Shuguang Hospital Affiliated to Shanghai University of Traditional Chinese Medicine, Shanghai, China; 2 Guangming Traditional Chinese Medicine Hospital Pudong New Area, Shanghai, China

**Keywords:** Mendelian randomization, gut microbiota, peripheral artery disease, causal effect, genetic association

## Abstract

Background: The causal relationship between gut microbiota and peripheral artery disease (PAD) is still not clear. In this research, we employed the Mendelian randomization (MR) technique to explore the potential causal connection between 211 gut microbiota species and PAD. We also investigated whether the causal effects operate in both directions.

Methods: We used Genome-wide Association Studies (GWAS) summary statistics data from the MiBioGen and FinnGen consortia to conduct a two-sample MR analysis to explore the causal link between gut microbiota and PAD. Sensitivity analysis is conducted to assess the robustness of the MR results. In addition to that, reverse MR analysis was performed to examine the inverse causal relationship.

Results: The inverse variance weighted (IVW) method provided evidence supporting a causal relationship between 9 specific gut microbiota taxa and PAD. The study findings indicated that family Family XI (OR=1.11, CI 1.00-1.24, P=0.048), genus *Lachnoclostridium* (OR=1.24, 1.02-1.50, P=0.033), and genus *Lachnospiraceae* UCG001 (OR=1.17, 1.01-1.35, P=0.031) are risk factors associated with PAD. class *Actinobacteria* (OR=0.84, 0.72-0.99, P=0.034), family *Acidaminococcaceae* (OR=0.80, 0.66-0.98, P=0.029), genus *Coprococcus2* (OR=0.79, 0.64-0.98, P=0.029), genus *Ruminococcaceae* UCG004 (OR=0.84, 0.72-0.99, P=0.032), genus *Ruminococcaceae* UCG010 (OR=0.74, 0.58-0.96, P=0.022), and order NB1n (OR=0.88, 0.79-0.98, P=0.02) may be associated with the risk factors of PAD. Moreover, our analysis did not uncover any evidence of a reverse causal relationship between PAD and the nine specific gut microbiota taxa investigated.

Conclusions: Our MR research has confirmed the potential causal relationship between gut microbiota and PAD while also identifying specific gut bacterial communities associated with PAD.

## INTRODUCTION

Peripheral arterial disease (PAD) is primarily seen as a manifestation of systemic atherosclerosis, presenting as chronic arterial occlusive disease in the lower extremities [1]. It is considered the third most prevalent manifestation of atherosclerotic vascular disease, with coronary artery disease (CAD) and stroke being the two leading presentations [2]. PAD can lead to intermittent claudication (IC), chronic limb-threatening ischemia (CLTI), limb functional impairment, and even life-threatening cardiovascular events [3, 4], and this mainly includes IC and CLTI. IC is clearly more common, characterized by pain in the calf, thigh, or buttock muscles that can be induced by repeated exercise and relieved by rest. CLTI is characterized by rest pain or tissue loss in the lower limbs. Compared to CLTI, the natural history of IC is benign, and few IC patients progress to CLTI. The development of PAD is influenced by various risk factors, such as smoking, hypertension, hyperlipidemia, and diabetes [5]. PAD is a widespread condition that affects more than 200 million individuals across the globe [6], and the prevalence of PAD ranges from 3% to 10%. However, based on data from older individuals, the prevalence among the elderly population can be as high as 15% to 20%. Furthermore, the current prevalence rates are even higher [7]. It has been found that the incidence of PAD increases with age, obesity, and diabetes [8]. PAD imposes a significant burden on affected individuals, families, and even the entire society. Therefore, it underscores the necessity of identifying risk factors associated with its development.

While the exact cause and underlying mechanisms of PAD remain incompletely understood, it is generally acknowledged to be a multifactorial disease with various contributing factors. The human gastrointestinal system provides a home to trillions of bacteria and archaea, which coexist with the human body in a mutually beneficial relationship that is crucial for human health [9]. Intestinal microbiota plays a role in various physiological functions, including maintaining metabolic stability, regulating immune responses, and resisting infections [10]. A growing body of research has established a link between the gut microbiota and the development and advancement of diseases, resulting in its acknowledgment as an endocrine organ [11]. Many studies and evidence suggest that the gut microbiota and its metabolites are associated with the formation and development of atherosclerosis (AS), including the formation and progression of atherosclerotic plaques, endothelial dysfunction, and thrombus formation [12, 13]. Atherosclerotic plaques themselves are a microbiota environment, containing microorganisms such as *Streptococcus, Pseudomonas, Klebsiella, Veillonella*, and *Chlamydia pneumoniae* [14]. A meta-analysis has revealed that shifts in the gut microbiota composition are linked to changes in the levels of bacterial metabolites, some of which have been shown to have atherosclerotic effects on endothelial cells [15]. In cross-sectional studies, it has been shown that symptomatic AS patients have higher abundances of *Collinsella, Enterobacteriaceae, Streptococcaceae*, and *Klebsiella* in their gut microbiota compared to healthy controls. On the other hand, the abundances of bacteria that produce short-chain fatty acids (SCFA) such as *Faecalibacterium, Roseburia*, and *Ruminococcaceae* are lower in symptomatic AS patients [16]. Additionally, there has been extensive research into the functions of specific metabolites originating from the gut microbiota, including trimethylamine N-oxide (TMAO) from carnitine metabolism, phenylacetylglutamine from phenylalanine metabolism, and bile acids from lipid metabolism, particularly in relation to AS. Most studies have been unable to establish a direct link between the composition of the gut microbiota and AS-related plaque vulnerability, rupture, or cardiovascular events. Currently, there is a lack of evidence for causal factors in this regard.

Although there have been rapid advancements in genomics, metabolomics, and other technologies that have revealed a strong correlation between gut microbiota and PAD, the exact causal relationship between them remains unclear [17, 18]. This method identifies and measures the causal impact of exposures on outcomes through the use of genetic variants as instrumental variables (IVs) [19, 20]. Because alleles are randomly distributed from parents to offspring, undergo independent assortment, and maintain genetic stability after birth, Mendelian randomization (MR), akin to randomized controlled trials (RCTs), can mitigate biases arising from conventional confounding factors (e.g., environmental influences, population traits, and dietary behaviors) and reverse causation [21, 22]. In this research, a two-sample MR analysis was conducted, leveraging summary statistics data from the Genome-wide Association Studies (GWAS) of the MiBioGen and FinnGen consortia, to explore the causal connection between gut microbiota and PAD.

## MATERIALS AND METHODS

### Research design

**Figure 1:**
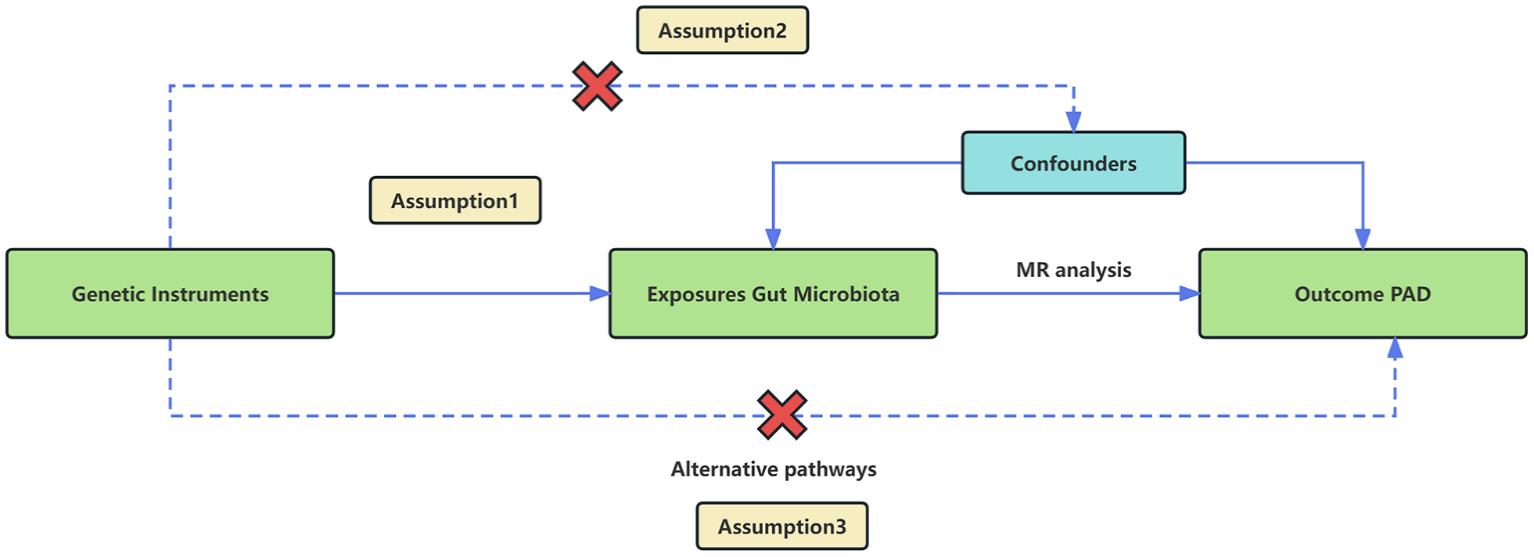
An overview of the MR analysis and its three primary assumptions.

We employed a two-sample MR design using GWAS meta-analysis data to furnish strong support for the causal link between gut microbiota and PAD. The study design and reporting of this study are in accordance with STROBE-MR guidelines [23]. To mitigate the impact of confounding variables on the outcomes, the MR approach must adhere to three essential assumptions (Figure 1). (1) Choose SNPs that exhibit significant associations with gut microbiota as IVs; (2) IVs should be independent, meaning they should not be correlated with other confounding factors like age and smoking; (3) IVs should solely be linked to the outcome via their connection with the exposure and must not affect the outcome through any alternative pathways [24].

### Data source

Genetic information pertaining to the gut microbiota is derived from an extensive case-control GWAS meta-analysis carried out by the international MiBioGen consortium [25]. The study includes 25 cohorts from countries such as the United States, Italy, and South Korea, with a total of 18,340 participants. The primary objective is to pinpoint genetic loci that impact the gut microbiota’s relative abundance through the examination of 16S rRNA sequencing profiles of the participants. GWAS summary statistics pertaining to PAD were extracted from the FinnGen Consortium R6 release dataset, comprising 9,021 PAD cases and 244,907 individuals serving as controls [26].

### Selection of IVs

Employ the subsequent quality control techniques to carefully choose suitable genetic IVs, thereby safeguarding the integrity and precision of causal inferences regarding the connection between gut microbiota composition and PAD risk. (1) In accordance with the typical practice observed in most MR investigations involving gut microbiota [27, 28], we established the significance threshold at P < 1.0×10^(-5) to detect an ample quantity of potential instrumental variables. This decision takes into account that the number of loci identified for gut microbiota is relatively limited [29]; (2) SNP variants associated with each bacterial taxonomic group are grouped together, and only independent SNPs are retained. The threshold for linkage disequilibrium (LD) aggregation is defined as r^2 < 0.001, and this aggregation is performed over a distance of 10,000 kb [30]; (3) In cases where palindrome SNPs are detected, allele frequency data is utilized to deduce the alleles on the forward strand; (4) An F-statistic threshold of >10 is set as the threshold for strong IVs. Otherwise, if the IVs are found to have a weak association with the exposure, they are considered to be excluded [31]. (5) To exclude SNPs linked to potential confounding factors like smoking and occupational exposure, we employed PhenoScanner to identify all SNPs meeting the specified criteria [32].

### Reverse MR data

The data source for reverse MR corresponds to that used for forward MR. In this scenario, we regard PAD as the exposure and extract SNPs closely linked to PAD as the exposure (p < 5*10^-8). Similar to forward MR, we also conducted a selection process in reverse MR, which includes eliminating linkage disequilibrium, palindromic sequences, and weakly correlated variables, and excluding SNPs with confounding factors. We employed the significant genera identified from the forward MR analysis as our results and subsequently conduct a two-sample MR analysis to establish the causal connection between PAD and gut microbiota.

### MR analysis

In this study, the primary analytical approach used to establish causal relationships was the inverse variance weighted (IVW) method [33]. This method calculates weighted averages based on the reciprocals of variances, under the assumption that all instrumental variables are valid. The results of this method are deemed the most accurate when there is neither heterogeneity nor horizontal pleiotropy. Furthermore, Supplementary Methods including MR-Egger regression, weighted median (WM), simple mode (SM), and weighted mode estimation are utilized alongside the IVW results [34]. MR-Egger has strong applicability for testing the hypothesis and can tolerate heterogeneity in more than 50% of the SNPs [35]. Weighted median is the median of the distribution function obtained by weighting all individual SNP effect values according to their weights. Robust estimation of weighted median can be obtained when at least 50% of the information comes from valid instrumental variables [36]. The principle of simple mode is to estimate causal effects directly using the effect estimate of the instrumental variable when there is only one instrumental variable available [37]. The weighted mode method is a commonly used statistical method for calculating the mode of a set of data. By considering the weight of each value, it can provide a more accurate description of the central tendency of the dataset [38].

### Horizontal pleiotropy and heterogeneity evaluation

To evaluate the robustness of the findings, additional sensitivity analyses were carried out. Heterogeneity among the instrumental variables (IVs) in the IVW method was quantified using Cochran’s Q statistic and MR-Egger regression (p < 0.05 was regarded as potential heterogeneity in IVs) [39]. The MR-Egger intercept test was applied to detect the presence of horizontal pleiotropy. If the p-value is greater than 0.05, it suggests the absence of horizontal pleiotropy [40]. Additionally, a powerful method known as MR pleiotropy residual sum and outlier (MR-PRESSO) is used to detect and remove potential horizontal pleiotropy outliers, which could greatly impact the estimation results within the MR-PRESSO framework. For each SNP, the MR-PRESSO outlier test calculates a p-value for its horizontal pleiotropy significance, while the MR-PRESSO global test calculates a p-value for the overall level of horizontal pleiotropy. Based on the p-values from the MR-PRESSO outlier test, SNPs are sorted in ascending order and sequentially removed from the list. Each time a SNP is removed from the list, the remaining SNPs are subjected to the MR-PRESSO global test. This recursive process is repeated until the p-value from the global test becomes insignificant (P > 0.05). The remaining SNPs after removing the ones with horizontal pleiotropy are then used for subsequent MR analysis [41]. We employ the “leave-one-out” method to assess sensitivity. This involves systematically removing each instrumental SNP one at a time and then re-performing the IVW analysis to determine if the causal estimates might be biased or influenced by a single SNP [42]. The statistical analysis was conducted using R version 4.3.1, with the “TwoSampleMR” R software package, which is widely used for this type of analysis.

### Data availability

The datasets used and/or analyzed during the current study are available from the corresponding author on reasonable request.

## RESULTS

### Causal effect of gut microbiota on PAD

**Figure 2:**
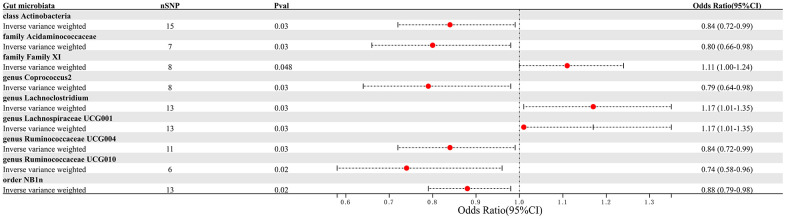
Forest plot summarizing the causal impact of gut microbiota composition on the risk of PAD, based on the IVW method.

This study selected 211 relative abundances of intestinal microbiota from gut microbiota GWAS data involving 18,340 participants as the exposure variable for the research. Due to the negative results of heterogeneity and pleiotropy tests, the IVW analysis results were used as the primary reference indicator in the study. The MR analysis results indicated that there may have been associations between nine different intestinal microbiota (1 class, 1 order, 2 families, and 5 genera) and PAD (Figure 2). Additionally, the results of the other four computational methods were found in Table 1. Lastly, the results of the five MR models analyzing the association between all gut microbiota and PAD risk were found in Supplementary Table 1.

**Table 1:** Statistical results of the four models other than IVW.

**Gut microbiota**	**Analytical methods**	**nSNP**	***P*-value**	**Odds ratio (95%CI)**
class *Actinobacteria*				
	MR Egger	15	0.45	1.20 (0.76-1.90)
	Weighted median	15	0.35	0.90 (0.72-1.12)
	Simple mode	15	0.22	0.76 (0.50-1.16)
	Weighted mode	15	0.93	0.99 (0.74-1.31)
family *Acidaminococcaceae*				
	MR Egger	7	0.33	0.72 (0.39-1.31)
	Weighted median	7	0.05	0.77 (0.60-1.00)
	Simple mode	7	0.19	0.74 (0.50-1.10)
	Weighted mode	7	0.14	0.73 (0.52-1.04)
family Family XI				
	MR Egger	8	0.21	1.63 (0.83-3.21)
	Weighted median	8	0.07	1.14 (0.99-1.30)
	Simple mode	8	0.19	1.20 (0.94-1.53)
	Weighted mode	8	0.21	1.19 (0.93-1.53)
genus *Coprococcus2*				
	MR Egger	8	0.48	1.89 (0.36-10.01)
	Weighted median	8	0.1	0.79 (0.60-1.04)
	Simple mode	8	0.11	0.64 (0.39-1.04)
	Weighted mode	8	0.1	0.65 (0.41-1.02)
genus *Lachnoclostridium*				
	MR Egger	13	0.43	1.30 (0.70-2.42)
	Weighted median	13	0.08	1.19 (0.98-1.44)
	Simple mode	13	0.24	1.22 (0.89-1.68)
	Weighted mode	13	0.31	1.19 (0.86-1.63)
genus *Lachnospiraceae* UCG001				
	MR Egger	13	0.43	1.30 (0.70-2.42)
	Weighted median	13	0.08	1.19 (0.98-1.44)
	Simple mode	13	0.24	1.22 (0.89-1.68)
	Weighted mode	13	0.31	1.19 (0.86-1.63)
genus *Ruminococcaceae* UCG004				
	MR Egger	11	0.52	1.35 (0.56-3.27)
	Weighted median	11	0.3	0.89 (0.71-1.11)
	Simple mode	11	0.67	0.92 (0.63-1.35)
	Weighted mode	11	0.72	0.93 (0.63-1.37)
genus *Ruminococcaceae* UCG010				
	MR Egger	6	0.32	0.64 (0.30-1.37)
	Weighted median	6	0.02	0.68 (0.50-0.93)
	Simple mode	6	0.09	0.64 (0.42-0.97)
	Weighted mode	6	0.09	0.66 (0.45-0.98)
order NB1n				
	MR Egger	13	0.56	0.87 (0.56-1.36)
	Weighted median	13	0.02	0.85 (0.74-0.98)
	Simple mode	13	0.2	0.86 (0.69-1.07)
	Weighted mode	13	0.2	0.86 (0.69-1.07)

**Figure 3:**
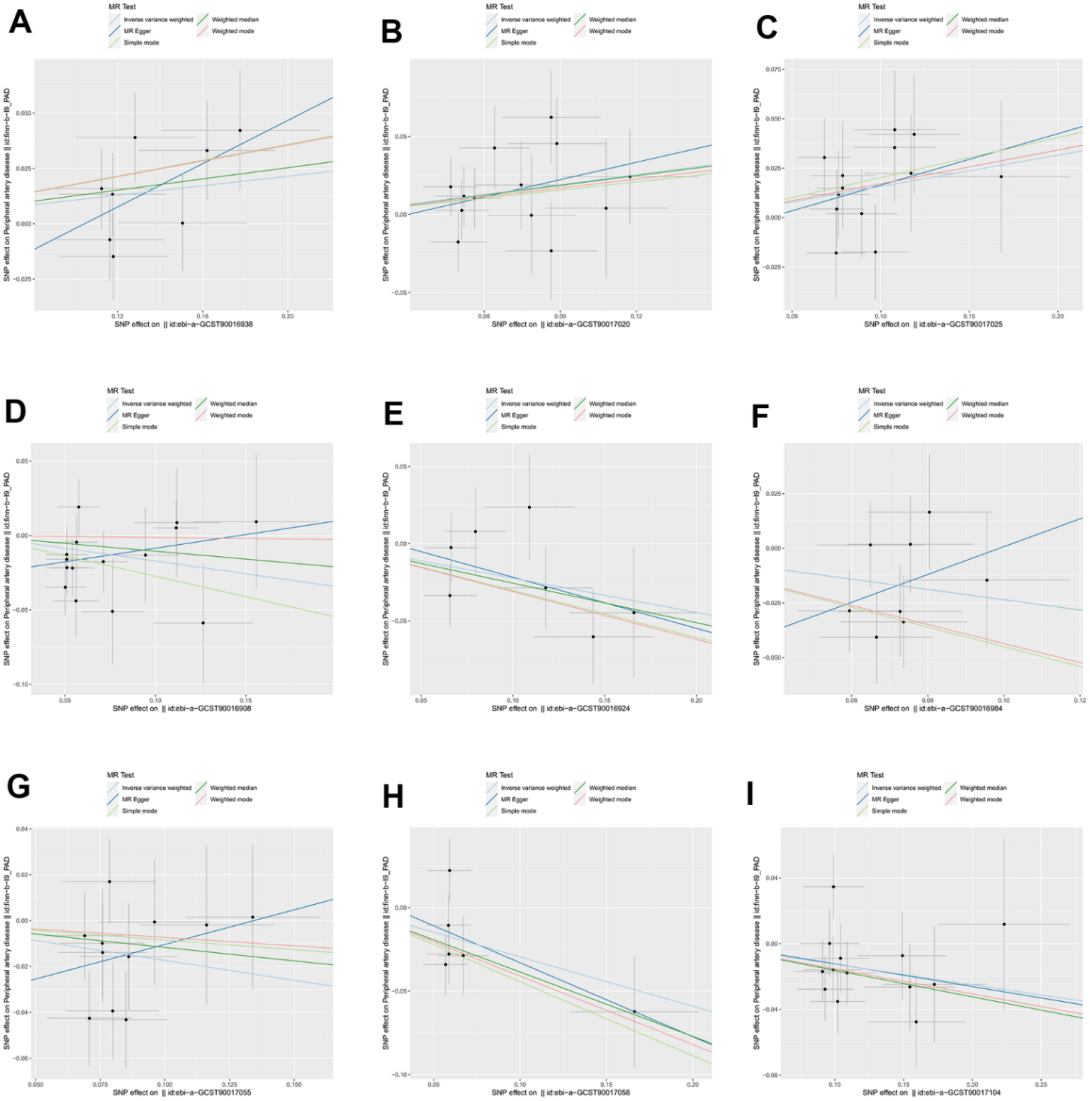
**The scatter plots for association between gut microbiota and PAD.** (**A**) family Family XI; (**B**) genus *Lachnoclostridium*; (**C**) genus *Lachnospiraceae*; (**D**) class *Actinobacteria*; (**E**) family *Acidaminococcaceae*; (**F**) genus *Coprococcus2*; (**G**) genus *Ruminococcaceae* UCG004; (**H**) genus *Ruminococcaceae* UCG010; (**I**) order NB1n. Note: SNP effects were plotted into lines for the inverse-variance weighted test (light blue line), MR-Egger (blue line), weighted median (green line), Simple mode (light green line) and Weighted mode (red line). The slope of the line corresponded to the causal estimation.

**Figure 4:**
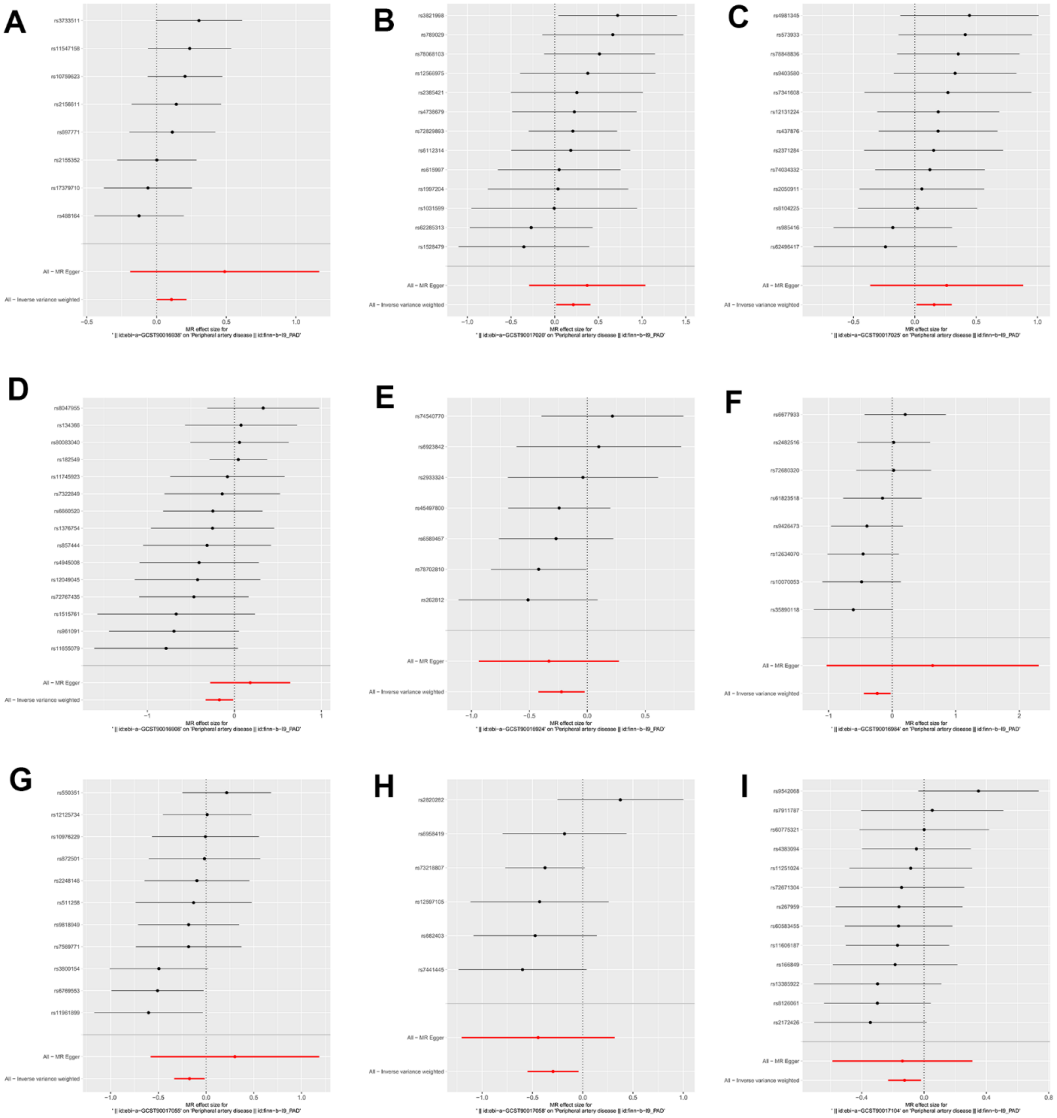
**The forest plots for the association between gut microbiota and PAD.** (**A**) family Family XI; (**B**) genus *Lachnoclostridium*; (**C**) genus *Lachnospiraceae*; (**D**) class *Actinobacteria*; (**E**) family *Acidaminococcaceae*; (**F**) genus *Coprococcus2*; (**G**) genus *Ruminococcaceae* UCG004; (**H**) genus *Ruminococcaceae* UCG010; (**I**) order NB1n.

We identified 3 microbiota taxonomic groups that were positively correlated with PAD. These included the family Family XI (OR=1.11, CI 1.00-1.24, P=0.048), the genus *Lachnoclostridium* (OR=1.24, CI 1.02-1.50, P=0.033), and the genus *Lachnospiraceae* UCG001 (OR=1.17, CI 1.01-1.35, P=0.031). Additionally, we found 6 microbiota taxonomic groups that were negatively correlated with PAD. These included the class *Actinobacteria* (OR=0.84, CI 0.72-0.99, P=0.034), the family *Acidaminococcaceae* (OR=0.80, CI 0.66-0.98, P=0.029), the genus *Coprococcus2* (OR=0.79, CI 0.64-0.98, P=0.029), the genus *Ruminococcaceae* UCG004 (OR=0.84, CI 0.72-0.99, P=0.032), the genus *Ruminococcaceae* UCG010 (OR=0.74, CI 0.58-0.96, P=0.022), and the order NB1n (OR=0.88, CI 0.79-0.98, P=0.02). We presented these statistical results using scatter plots (Figure 3) and forest plots (Figure 4) to illustrate the causal effects of each SNP of the gut microbiota on PAD risk.

To assess heterogeneity in the results, a Cochran’s Q test with a distribution set at 10,000 for both the IVW and MR-Egger regression analyses was conducted. The test results indicated the absence of heterogeneity in the SNPs for each microbiota taxonomic group. Additionally, the symmetry of the funnel plot suggested that there was no heterogeneity in the study (Supplementary Figure 1). The P-values of the intercept term in the MR-Egger regression consistently exceeded 0.05, signifying the absence of horizontal pleiotropy. In addition, in the MR-PRESSO analysis, if the global test p-value for the associated microbiota community is greater than 0.05, it indicates the absence of horizontal pleiotropy (Table 2). The leave-one-out test results confirmed the robustness of the MR findings in the study (Supplementary Figure 2).

**Table 2:** Heterogeneity and level of pleiotropy testing for 9 specific microbiota communities.

**Gut microbiota**	**nsnp**	**Q_inverse.variance.weighted**	**Q_pval_inverse.variance.weighted**	**Q_MR.egger**	**Q_pval_MR.egger**	**Egger_intercept**	**Egger_intercept_pval**	**MR-PRESSO_global_test_p**
family Family XI	8	6.51	0.48	5.26	0.51	-0.05	0.31	0.523
genus *Lachnoclostridium*	13	9.01	0.7	8.77	0.64	-0.01	0.63	0.735
genus *Lachnospiraceae* UCG001	13	7.15	0.85	7.04	0.8	-0.01	0.75	0.825
class *Actinobacteria*	15	12.48	0.57	9.89	0.7	-0.03	0.13	0.676
family *Acidaminococcaceae*	7	4.9	0.56	4.76	0.45	0.01	0.72	0.588
genus *Coprococcus2*	8	6.36	0.5	5.3	0.51	-0.06	0.34	0.53
genus *Ruminococcaceae* UCG004	11	9.58	0.48	8.43	0.49	-0.04	0.31	0.49
genus *Ruminococcaceae* UCG010	6	6.04	0.3	5.8	0.21	0.01	0.7	0.376
order NB1n	13	10.31	0.59	10.31	0.5	0	0.95	0.528

### Reverse-direction MR analyses

We employed the IVW method to perform a reverse MR analysis, aiming to examine the causal association between 9 specific gut microbiota taxa and PAD. Three SNPs that were closely associated with PAD were identified. As shown in Table 3, none of the gut microbiota taxa categories, including class *Actinobacteria* (P=0.31), family *Acidaminococcaceae* (P=0.88), family Family XI (P=0.99), genus *Coprococcus2* (P=0.93), genus *Lachnoclostridium* (P=0.14), genus *Lachnospiraceae* UCG001 (P=0.77), genus *Ruminococcaceae* UCG004 (P=0.66), genus *Ruminococcaceae* UCG010 (P=0.53), and order NB1n (P=0.72), showed a significant reverse causal relationship with PAD. The reliability of our findings was validated through the utilization of the MR-Egger regression method and the Cochran’s Q test.

**Table 3:** Reverse MR analysis results for 9 specific microbiota communities.

**Gut microbiota**	**OR (95%CI)**	**Pval**	**Q_pval_inverse.variance.weighted**	**Q_pval_MR.egger**	**Egger_intercept (pval)**
class *Actinobacteria*	0.95 (0.87-1.05)	0.31	0.6	0.78	0.09 (0.51)
family *Acidaminococcaceae*	0.99 (0.89-1.10)	0.88	0.99	0.89	-0.01 (0.96)
family Family XI	1.00 (0.79-1.28)	0.99	0.27	0.46	-0.3 (0.38)
genus *Coprococcus2*	1.00 (0.89-1.11)	0.93	0.88	0.78	-0.05 (0.74)
genus *Lachnoclostridium*	1.08 (0.98-1.19)	0.14	0.3	0.12	-0.02 (0.92)
genus *Lachnospiraceae* UCG001	0.98 (0.85-1.13)	0.77	0.22	0.52	-0.18 (0.35)
genus *Ruminococcaceae* UCG004	0.95 (0.77-1.18)	0.66	0.05	0.01	0.02 (0.96)
genus *Ruminococcaceae* UCG010	0.97 (0.87-1.07)	0.53	0.97	0.82	0 (0.98)

## DISCUSSION

### Main findings and interpretation

For the first time, this study evaluated the possible causal connection between gut microbiota and PAD utilizing bidirectional MR. We identified specific gut microbiota communities strongly linked to PAD at various taxonomic levels, including phylum, order, family, and genus. Among them, family Family XI, genus *Lachnoclostridium*, and genus *Lachnospiraceae* UCG001 were identified as potential risk factors for PAD, while class *Actinobacteria, family Acidaminococcaceae*, genus *Coprococcus2*, genus *Ruminococcaceae* UCG004, genus *Ruminococcaceae* UCG010, and order NB1n were identified as potential protective factors for PAD. Sensitivity analysis indicated the absence of heterogeneity or horizontal pleiotropy, affirming that our MR analysis remains unaffected by confounding factors. Furthermore, the “leave-one-out” analysis validated the study’s robustness.

The gut microbiota, as the most complex microbiota ecosystem in the human body, regulates a wide range of physiological processes [43]. Simultaneously, progress in high-throughput DNA sequencing technology has furnished us with a more profound comprehension of the gut microbiota’s composition and functionalities. Generally, the gut microbiota community comprises a diverse assortment of bacteria present in specific proportions. These bacteria interact, constrain, and depend on each other, establishing a qualitative and quantitative ecological balance [44]. Gut dysbiosis increases intestinal permeability by inhibiting tight junction proteins, allowing translocation of lipopolysaccharides into the circulation [45]. The lipopolysaccharides derived from gut dysbiosis bind to Toll-like receptors (TLRs) and activate downstream immune responses [46]. Lipopolysaccharides bind to the TLR4 complex and its co-receptor, cluster of differentiation 14 (CD14). Upregulation of TLRs initiates the inflammation-driven process of atherosclerosis [47]. The interaction between lipopolysaccharides and TLR4 activates the MYD88 and NFκB pathways, thereby enhancing the synthesis of pro-inflammatory cytokines such as IL-6, IL-1, IL-27, and TNF-α. These inflammatory cytokines play a role in the development of atherosclerosis and cardiovascular diseases [48].

The primary characteristic of PAD is AS. A growing body of evidence underscores the pivotal role of the gut microbiota in the initiation and advancement of AS, rendering it a novel target for the prevention and management of AS [49]. During the early 21st century, numerous investigations documented the presence of bacterial DNA from diverse species within atherosclerotic plaques, representing the initial evidence of a connection between the microbiota and AS [50, 51]. The gut microbiota can serve as a circulating factor that promotes or prevents the formation of AS by generating metabolites from dietary sources [52, 53]. Gut dysbiosis, which signifies the perturbation of gut microbiota linked to various diseases, was also highlighted. Another study revealed that individuals with AS display an ecological imbalance characterized by an overabundance of *Actinobacteria* in their gut compared to a healthy control group, a phenomenon associated with butyrate production [54]. Furthermore, changes in the composition of gut microbiota and the presence of its byproducts, including short-chain fatty acids (SCFAs) and bile acids (BAs), are linked to the risk of AS development [55, 56].

Particular bacterial species within the gut are correlated with the progression of PAD. At the phylum level, significant alterations in the gut microbiota composition are observed in PAD patients compared to the control group [57]. In this study, we have identified and confirmed 9 specific gut microbiota communities that have a potential causal relationship with PAD. Dysbiosis has the potential to compromise the integrity of the intestinal barrier, resulting in the release of detrimental metabolites like lipopolysaccharides (LPS) and other bacterial constituents such as peptidoglycans into the bloodstream. This, in turn, can incite an inflammatory reaction and contribute to the progression of AS [58, 59]. A study has established a positive correlation between the presence of *Lachnoclostridium* and the production of inflammatory cytokines and LPS in the serum [60]. Trimethylamine (TMA) is a small-molecule byproduct resulting from the microbiota metabolism of choline, carnitine, and phosphatidylcholine within the gut [61, 62]. Within the liver, TMA undergoes conversion into trimethylamine-N-oxide (TMAO) through the action of flavin-containing monooxygenase 3 [63, 64]. A growing body of evidence supports the notion that plasma TMAO serves as an independent risk factor for AS. Research has shown that *Lachnoclostridium* strains can effectively convert choline into TMA. When co-administered with choline to ApoE-/- mice, these strains can increase the levels of TMAO in the serum and promote the formation of AS [65]. *Ruminococcaceae* generates acetate and butyrate, serving as the primary energy source for intestinal epithelial cells while concurrently suppressing the signaling pathways of pro-inflammatory cytokines [66]. The anti-atherosclerotic effects of butyrate have been extensively confirmed [67, 68]. Butyrate can reduce cholesterol levels in the blood by converting cholesterol to coprostanol [69]. In addition, *Coprococcus* can also produce butyrate [70]. In AS disease, the significant reduction of *Acidaminocccaceae*, decreased synthesis of SCFAs, and increased TMAO levels can lead to an increase in atherogenic lipoproteins in the blood, contributing to the development of AS [71].

### Limitation

However, this study also has limitations: (1) Human behavior is intricate, and while comprehending the genetic predisposition to a disease can aid in its prevention to a certain degree [72], environmental factors also contribute to the disease’s onset [73]. MR can only partially address confounding factors, including environmental influences. (2) The study’s outcome data are based on European and American populations, and further validation is required to ascertain whether the results are representative of the broader population. (3) While confirming a causal link between gut microbiota and PAD, the precise underlying mechanisms remain elusive, necessitating additional research.

## CONCLUSIONS

The study obtained data from GWAS databases and employed a two-sample bidirectional MR approach to validate the potential causal connection between gut microbiota and PAD. These findings offer novel insights into the pathogenesis of PAD and potential treatment strategies. Subsequent studies should aim to delve deeper into the mechanisms through which these microbiota communities impact PAD and investigate potential therapeutic approaches targeting the gut microbiota. Additionally, gut microbiota may serve as a novel important predictive biomarker for PAD.

## Supplementary Material

Supplementary Figures

Supplementary Table 1
