# Supplementary material for: Genetic support of the causal association between gut microbiota and peripheral artery disease: a bidirectional Mendelian randomization study
Source: Aging (Albany NY). 2024 Jan 9;16(1):762–78. doi: 10.18632/aging.205417 (PMC10817407; doi:10.18632/aging.205417)
Supplement: Supplementary Figures [file aging-16-205417-s001.pdf]

SUPPLEMENTARY FIGURES

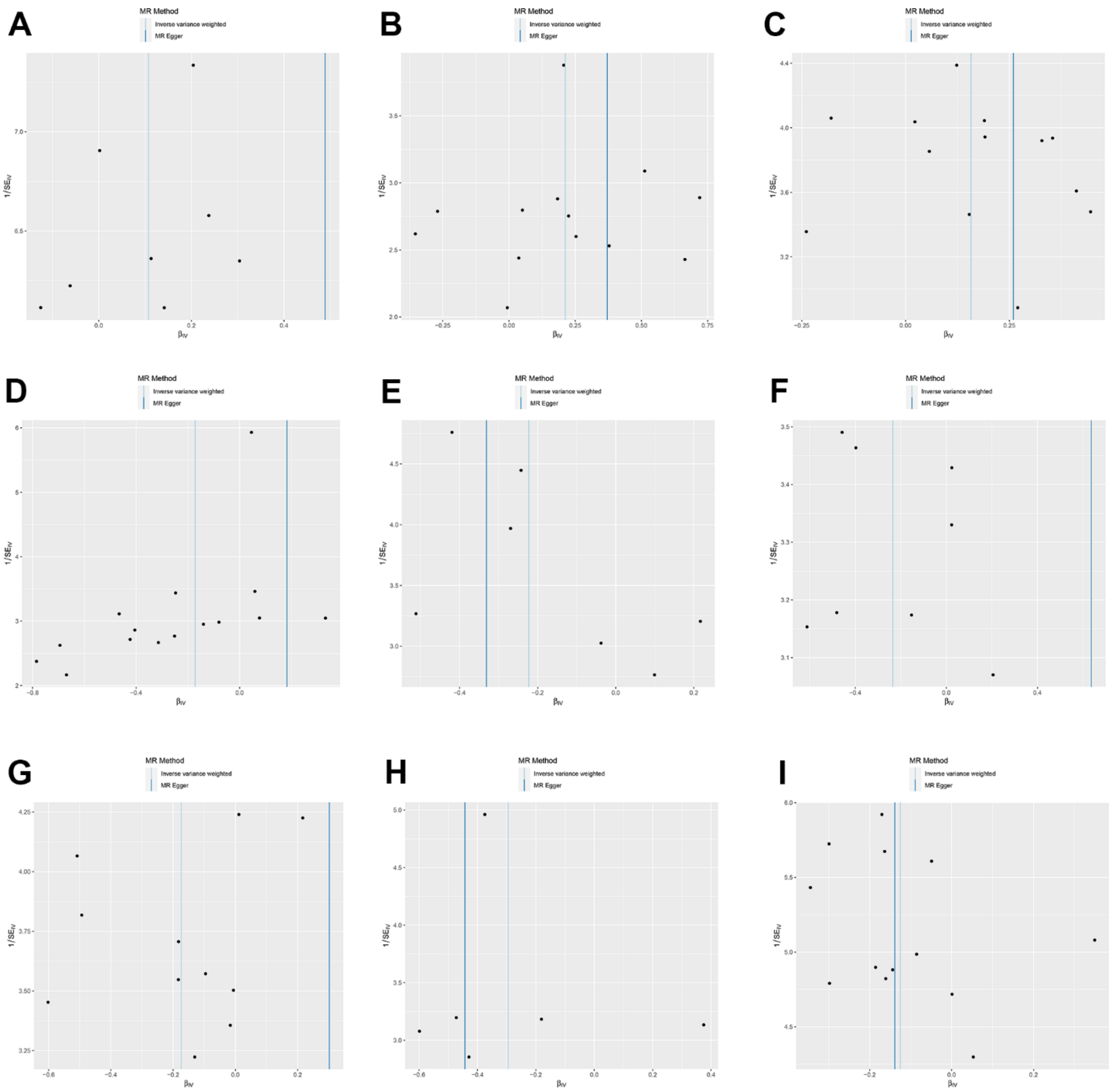

Supplementary Figure 1. The funnel plots for the association between gut microbiota and PAD. (**A**) family Family XI; (**B**) genus *Lachnoclostridium*; (**C**) genus *Lachnospiraceae* UCG001; (**D**) class *Actinobacteria*; (**E**) family *Acidaminococcaceae*; (**F**) genus *Coprococcus2*; (**G**) genus *Ruminococcaceae* UCG004; (**H**) genus *Ruminococcaceae* UCG010; (**I**) order NB1n.

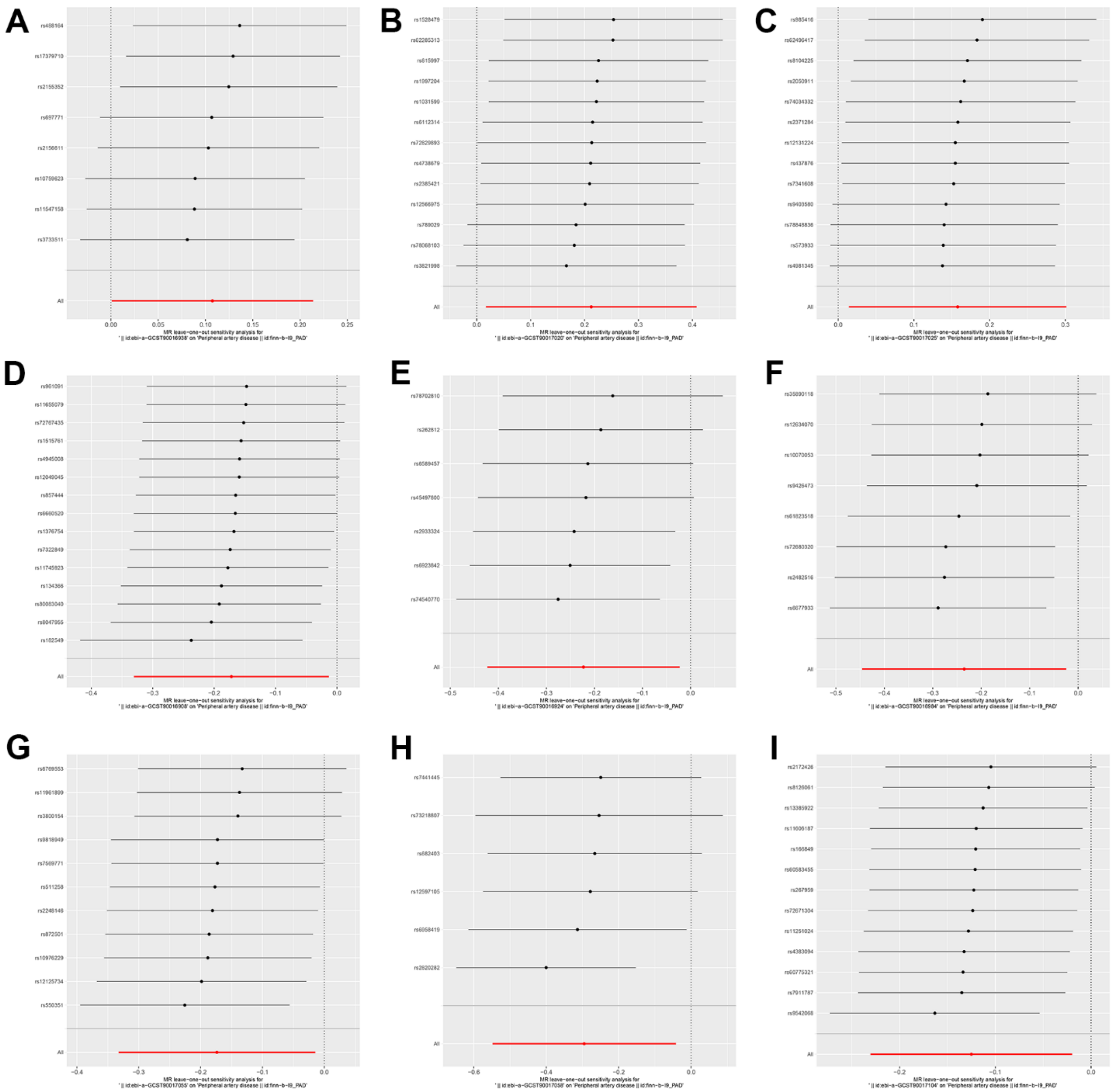

Supplementary Figure 2. The leave-one-out sensitivity analysis for the association between gut microbiota and PAD. (**A**) family Family XI; (**B**) genus *Lachnoclostridium*; (**C**) genus *Lachnospiraceae* UCG001; (**D**) class *Actinobacteria*; (**E**) family *Acidaminococcaceae*; (**F**) genus Coprococcus2; (**G**) genus *Ruminococcaceae* UCG004; (**H**) genus *Ruminococcaceae* UCG010; (**I**) order NB1n.
