# Supplementary material for: Genetic support of the causal association between gut microbiota and peripheral artery disease: a bidirectional Mendelian randomization study
Source: Aging (Albany NY). 2024 Jan 9;16(1):762–78. doi: 10.18632/aging.205417 (PMC10817407; doi:10.18632/aging.205417)
Supplement: Supplementary Table 1 [file aging-16-205417-s002.docx]

**Supplementary Table 1. The results of the five MR models analyzing the association between all gut microbiota and PAD risk.**

|  | **id.exposure** | **nsnp** | **p_no** | **b_MR.Egger** | **se_MR.Egger** | **pval_MR.Egger** | **b_Weighted.median** | **se_Weighted.median** | **pval_Weighted.median** | **b_Inverse.variance.weighted** | **se_Inverse.variance.weighted** | **pval_Inverse.variance.weighted** | **Q_MR.Egger** | **Q_df_MR.Egger** | **Q_pval_MR.Egger** | **Q_Inverse.variance.weighted** | **Q_df_Inverse.variance.weighted** | **Q_pval_Inverse.variance.weighted** | **egger_intercept** | **se** | **pval** |
| --- | --- | --- | --- | --- | --- | --- | --- | --- | --- | --- | --- | --- | --- | --- | --- | --- | --- | --- | --- | --- | --- |
| 1 | Gut microbiota abundance (class Actinobacteria id.419) | 15 | MR Egger Weighted median | 0.182615343 | 0.23452362 | 0.450118768 | -0.105848997 | 0.112327499 | 0.34602633 | -0.171867238 | 0.08100647 | 0.033867286 | 9.88655312 | 13 | 0.703183971 | 12.4806854 | 14 | 0.567758829 | -0.026810699 | 0.016646084 | 0.131262981 |
| 2 | Gut microbiota abundance (class Alphaproteobacteria id.2379) | 7 | MR Egger Weighted median Inverse variance weighted | -0.081567251 | 0.366694211 | 0.832772599 | 0.078138372 | 0.128083485 | 0.541823299 | 0.175790974 | 0.097839757 | 0.072379554 | 2.857098351 | 5 | 0.722005662 | 3.387422483 | 6 | 0.758882371 | 0.024233818 | 0.033277535 | 0.499131638 |
| 3 | Gut microbiota abundance (class Bacilli id.1673) | 18 | MR Egger Weighted median Inverse variance weighted | 0.013698382 | 0.243447914 | 0.955824813 | 0.12025478 | 0.118630737 | 0.310730745 | 0.004717992 | 0.086000709 | 0.956250062 | 18.50416739 | 16 | 0.295211127 | 18.50598165 | 17 | 0.357613403 | -0.000709287 | 0.017908007 | 0.968896273 |
| 4 | Gut microbiota abundance (class Bacteroidia id.912) | 14 | MR Egger Weighted median Inverse variance weighted | 0.208130093 | 0.188290682 | 0.290670555 | 0.09062799 | 0.118832996 | 0.445672127 | 0.130107476 | 0.092264256 | 0.158492085 | 6.972701443 | 12 | 0.85941227 | 7.198661847 | 13 | 0.891625359 | -0.007055363 | 0.014842368 | 0.643070982 |
| 5 | Gut microbiota abundance (class Betaproteobacteria id.2867) | 11 | MR Egger Weighted median Inverse variance weighted | 0.182726587 | 0.352496539 | 0.616696684 | -0.005987107 | 0.139761744 | 0.965830712 | -0.057313943 | 0.105799545 | 0.588010021 | 6.511306357 | 9 | 0.687852542 | 7.020941242 | 10 | 0.723466504 | -0.01710216 | 0.023956391 | 0.493393577 |
| 6 | Gut microbiota abundance (class Clostridia id.1859) | 12 | MR Egger Weighted median Inverse variance weighted | -0.064755862 | 0.26897171 | 0.814610317 | 0.08080899 | 0.131243598 | 0.538080935 | 0.024435349 | 0.098576733 | 0.804226092 | 7.148181977 | 10 | 0.711385536 | 7.275202162 | 11 | 0.776379248 | 0.006703596 | 0.018809249 | 0.728946756 |
| 7 | Gut microbiota abundance (class Coriobacteriia id.809) | 13 | MR Egger Weighted median Inverse variance weighted | 0.421998839 | 0.430067914 | 0.347564603 | 0.146630021 | 0.135179007 | 0.278050115 | 0.081714229 | 0.10137427 | 0.420205494 | 7.412472182 | 11 | 0.764765042 | 8.075355795 | 12 | 0.779214193 | -0.025138742 | 0.030876274 | 0.432815986 |
| 8 | Gut microbiota abundance (class Deltaproteobacteria id.3087) | 13 | MR Egger Weighted median Inverse variance weighted | 0.439749138 | 0.252562617 | 0.109514543 | 0.082806829 | 0.13490676 | 0.539342304 | 0.094808829 | 0.100592506 | 0.345934715 | 12.76294873 | 11 | 0.309100959 | 15.29254831 | 12 | 0.225826385 | -0.027195138 | 0.018418088 | 0.167843439 |
| 9 | Gut microbiota abundance (class Erysipelotrichia id.2147) | 13 | MR Egger Weighted median Inverse variance weighted | -0.860262742 | 0.458965255 | 0.087668191 | -0.013684954 | 0.13948508 | 0.921844533 | 0.023904398 | 0.105866523 | 0.821358906 | 4.664696412 | 11 | 0.946312113 | 8.584402105 | 12 | 0.737958051 | 0.055133712 | 0.027847775 | 0.073291265 |
| 10 | Gut microbiota abundance (class Gammaproteobacteria id.3303) | 6 | MR Egger Weighted median Inverse variance weighted | -0.476773686 | 0.479346624 | 0.376211597 | -0.109358096 | 0.197057662 | 0.578925575 | -0.182786958 | 0.149604002 | 0.221781275 | 4.831369394 | 4 | 0.305041719 | 5.341916026 | 5 | 0.375591793 | 0.02288097 | 0.035193451 | 0.551048377 |
| 11 | Gut microbiota abundance (class Lentisphaeria id.2250) | 8 | MR Egger Weighted median Inverse variance weighted | 0.187773836 | 0.289649692 | 0.540804736 | -0.081767544 | 0.083342619 | 0.326542838 | -0.024107539 | 0.079248792 | 0.760974983 | 8.818278109 | 6 | 0.184058862 | 9.672763288 | 7 | 0.207888397 | -0.031507442 | 0.041321602 | 0.474664226 |
| 12 | Gut microbiota abundance (class Melainabacteria id.1589) | 10 | MR Egger Weighted median Inverse variance weighted | -0.35366459 | 0.205895073 | 0.124179249 | 0.076004164 | 0.092407819 | 0.410800187 | -0.016000332 | 0.069190827 | 0.817121115 | 6.308258019 | 8 | 0.612744933 | 9.326731219 | 9 | 0.407676979 | 0.039208711 | 0.022567783 | 0.120524134 |
| 13 | Gut microbiota abundance (class Methanobacteria id.119) | 10 | MR Egger Weighted median Inverse variance weighted | -0.127169636 | 0.227713416 | 0.591810856 | -0.002712821 | 0.07278923 | 0.970270089 | 0.008005883 | 0.055482231 | 0.885266462 | 5.066778764 | 8 | 0.750415469 | 5.441404605 | 9 | 0.794258718 | 0.022495322 | 0.036753046 | 0.557484489 |
| 14 | Gut microbiota abundance (class Mollicutes id.3920) | 12 | MR Egger Weighted median Inverse variance weighted | -0.035500494 | 0.396350725 | 0.93039874 | -0.062783214 | 0.118297914 | 0.595611972 | -0.041406464 | 0.110426712 | 0.707684399 | 19.15653743 | 10 | 0.038318881 | 19.15700248 | 11 | 0.058332832 | -0.000538391 | 0.034554625 | 0.98787524 |
| 15 | Gut microbiota abundance (class Negativicutes id.2164) | 12 | MR Egger Weighted median Inverse variance weighted | 0.355217054 | 0.365118963 | 0.353557502 | 0.120393326 | 0.146042791 | 0.409729235 | 0.111583722 | 0.105775231 | 0.291464875 | 9.398166552 | 10 | 0.494778323 | 9.884209135 | 11 | 0.540831802 | -0.016081593 | 0.023067042 | 0.501583663 |
| 16 | Gut microbiota abundance (class Verrucomicrobiae id.4029) | 11 | MR Egger Weighted median Inverse variance weighted | -0.284588142 | 0.294773938 | 0.359545603 | 0.097834247 | 0.116209098 | 0.399854491 | 0.054441114 | 0.088140546 | 0.53679781 | 5.710304567 | 9 | 0.768525496 | 7.162991578 | 10 | 0.709973171 | 0.027944777 | 0.023185401 | 0.258830128 |
| 17 | Gut microbiota abundance (family Acidaminococcaceae id.2166) | 7 | MR Egger Weighted median | -0.331288701 | 0.30833723 | 0.331719349 | -0.255743564 | 0.131980884 | 0.052655944 | -0.222788606 | 0.102095945 | 0.029098507 | 4.758717251 | 5 | 0.446030554 | 4.897790042 | 6 | 0.556987706 | 0.01142151 | 0.030626858 | 0.724491318 |
| 18 | Gut microbiota abundance (family Actinomycetaceae id.421) | 5 | MR Egger Weighted median Inverse variance weighted | -0.122601336 | 0.231223045 | 0.63269118 | -0.100471308 | 0.126434155 | 0.426815254 | -0.101832496 | 0.101766559 | 0.316997055 | 0.673237111 | 3 | 0.879480057 | 0.68324333 | 4 | 0.95337917 | 0.002483228 | 0.024824558 | 0.926629644 |
| 19 | Gut microbiota abundance (family Alcaligenaceae id.2875) | 11 | MR Egger Weighted median Inverse variance weighted | 0.552962475 | 0.507258904 | 0.303993997 | -0.028460755 | 0.148403903 | 0.847915307 | -0.036871947 | 0.110651013 | 0.738962656 | 7.930485131 | 9 | 0.541166588 | 9.350110367 | 10 | 0.499230204 | -0.038385867 | 0.032216956 | 0.263936649 |
| 20 | Gut microbiota abundance (family Bacteroidaceae id.917) | 9 | MR Egger Weighted median Inverse variance weighted | 0.052504943 | 0.814910127 | 0.950428927 | -0.081456836 | 0.162388476 | 0.615936889 | -0.111757951 | 0.147430035 | 0.448426684 | 12.45158302 | 7 | 0.086652543 | 12.52664878 | 8 | 0.129207339 | -0.0108572 | 0.052851844 | 0.843084703 |
| 21 | Gut microbiota abundance (family Bacteroidales S24 7group id.11173) | 8 | MR Egger Weighted median Inverse variance weighted | -0.119506744 | 0.330761932 | 0.730251366 | 0.125018136 | 0.107944965 | 0.246796498 | 0.15827345 | 0.082408451 | 0.054782492 | 1.811478532 | 6 | 0.936194725 | 2.563453044 | 7 | 0.92224368 | 0.028159342 | 0.03247289 | 0.419175943 |
| 22 | Gut microbiota abundance (family Bifidobacteriaceae id.433) | 12 | MR Egger Weighted median Inverse variance weighted | 0.080693659 | 0.28126946 | 0.780052309 | 0.043636206 | 0.115380664 | 0.705286928 | 0.106242152 | 0.085546176 | 0.214263296 | 9.528348766 | 10 | 0.482799313 | 9.537440364 | 11 | 0.572410269 | 0.001978483 | 0.02074972 | 0.925920593 |
| 23 | Gut microbiota abundance (family Christensenellaceae id.1866) | 12 | MR Egger Weighted median Inverse variance weighted | 0.080693659 | 0.28126946 | 0.780052309 | 0.043636206 | 0.115380664 | 0.705286928 | 0.106242152 | 0.085546176 | 0.214263296 | 9.528348766 | 10 | 0.482799313 | 9.537440364 | 11 | 0.572410269 | 0.001978483 | 0.02074972 | 0.925920593 |
| 24 | Gut microbiota abundance (family Clostridiaceae1 id.1869) | 10 | MR Egger Weighted median Inverse variance weighted | -0.036650957 | 0.405359707 | 0.930179537 | 0.020175437 | 0.148837374 | 0.892174214 | 0.094113575 | 0.131943679 | 0.47566882 | 15.10601185 | 8 | 0.05711661 | 15.3286629 | 9 | 0.082294817 | 0.01020164 | 0.029708966 | 0.740157948 |
| 25 | Gut microbiota abundance (family Clostridiales vadin BB60 group id.11286) | 15 | MR Egger Weighted median Inverse variance weighted | 0.033256081 | 0.193012322 | 0.865854897 | 0.115259668 | 0.093700838 | 0.218666582 | 0.112954126 | 0.070585049 | 0.109541872 | 4.772567652 | 13 | 0.979864331 | 4.969390759 | 14 | 0.986234126 | 0.007797398 | 0.01757566 | 0.664591687 |
| 26 | Gut microbiota abundance (family Coriobacteriaceae id.811) | 13 | MR Egger Weighted median Inverse variance weighted | 0.421998839 | 0.430067914 | 0.347564603 | 0.146630021 | 0.135049417 | 0.27758923 | 0.081714229 | 0.10137427 | 0.420205494 | 7.412472182 | 11 | 0.764765042 | 8.075355795 | 12 | 0.779214193 | -0.025138742 | 0.030876274 | 0.432815986 |
| 27 | Gut microbiota abundance (family Defluviitaleaceae id.1924) | 11 | MR Egger Weighted median Inverse variance weighted | -0.436701308 | 0.331600693 | 0.220399617 | -0.059650779 | 0.109831858 | 0.587054079 | -0.065365137 | 0.098650695 | 0.507592051 | 13.75540966 | 9 | 0.131295659 | 15.85084637 | 10 | 0.103982442 | 0.039531307 | 0.033761319 | 0.271704302 |
| 28 | Gut microbiota abundance (family Desulfovibrionaceae id.3169) | 10 | MR Egger Weighted median Inverse variance weighted | 0.49056001 | 0.265346361 | 0.101670946 | 0.051588783 | 0.149699376 | 0.730383163 | 0.066866616 | 0.12228821 | 0.584519387 | 10.3801411 | 8 | 0.239351864 | 14.37797594 | 9 | 0.109500852 | -0.036237515 | 0.020644417 | 0.117279842 |
| 29 | Gut microbiota abundance (family Enterobacteriaceae id.3469) | 7 | MR Egger Weighted median Inverse variance weighted | 1.654847094 | 1.18155471 | 0.220244203 | -0.098773863 | 0.19080279 | 0.604684938 | 0.0116806 | 0.213373543 | 0.956343616 | 12.14477239 | 5 | 0.032857019 | 16.97765164 | 6 | 0.00936573 | -0.121473415 | 0.086116814 | 0.21744879 |
| 30 | Gut microbiota abundance (family Erysipelotrichaceae id.2149) | 13 | MR Egger Weighted median Inverse variance weighted | -0.860262742 | 0.458965255 | 0.087668191 | -0.013684954 | 0.14026327 | 0.922276767 | 0.023904398 | 0.105866523 | 0.821358906 | 4.664696412 | 11 | 0.946312113 | 8.584402105 | 12 | 0.737958051 | 0.055133712 | 0.027847775 | 0.073291265 |
| 31 | Gut microbiota abundance (family Family XI id.1936) | 8 | MR Egger Weighted median | 0.488632858 | 0.345547396 | 0.207067637 | 0.12668199 | 0.071029796 | 0.074504148 | 0.107343709 | 0.054310817 | 0.048101553 | 5.258846715 | 6 | 0.511068331 | 6.507255885 | 7 | 0.48191753 | -0.051046537 | 0.045686491 | 0.306589572 |
| 32 | Gut microbiota abundance (family Family XIII id.1957) | 9 | MR Egger Weighted median Inverse variance weighted | -0.442416652 | 0.465369949 | 0.373429499 | -0.054863073 | 0.161827968 | 0.734593939 | -0.083903721 | 0.122210354 | 0.492365304 | 2.721755408 | 7 | 0.909494561 | 3.359205629 | 8 | 0.909837836 | 0.023865386 | 0.029891335 | 0.450868874 |
| 33 | Gut microbiota abundance (family Lachnospiraceae id.1987) | 16 | MR Egger Weighted median Inverse variance weighted | -0.016720247 | 0.335818955 | 0.96099353 | -0.200671906 | 0.129370992 | 0.120869276 | -0.055025245 | 0.096438083 | 0.568287167 | 11.12223394 | 14 | 0.676407906 | 11.13641404 | 15 | 0.742865607 | -0.002396878 | 0.020128262 | 0.906903624 |
| 34 | Gut microbiota abundance (family Lactobacillaceae id.1836) | 8 | MR Egger Weighted median Inverse variance weighted | -0.01262572 | 0.205361116 | 0.952973295 | 0.112496033 | 0.104492757 | 0.281662718 | 0.050151297 | 0.076722468 | 0.513323416 | 7.521172981 | 6 | 0.275322192 | 7.660602548 | 7 | 0.363470419 | 0.008640634 | 0.025908087 | 0.750095284 |
| 35 | Gut microbiota abundance (family Methanobacteriaceae id.121) | 10 | MR Egger Weighted median Inverse variance weighted | -0.127169636 | 0.227713416 | 0.591810856 | -0.002712821 | 0.072449376 | 0.970130694 | 0.008005883 | 0.055482231 | 0.885266462 | 5.066778764 | 8 | 0.750415469 | 5.441404605 | 9 | 0.794258718 | 0.022495322 | 0.036753046 | 0.557484489 |
| 36 | Gut microbiota abundance (family Oxalobacteraceae id.2966) | 14 | MR Egger Weighted median Inverse variance weighted | -0.251640241 | 0.197413793 | 0.226550538 | -0.053955577 | 0.074110396 | 0.466587085 | 0.003834067 | 0.051131475 | 0.940227063 | 10.48718994 | 12 | 0.573300086 | 12.28232502 | 13 | 0.504640253 | 0.035747117 | 0.026680407 | 0.20512228 |
| 37 | Gut microbiota abundance (family Pasteurellaceae id.3689) | 14 | MR Egger Weighted median Inverse variance weighted | 0.005291576 | 0.137780148 | 0.969995567 | -0.012749315 | 0.08883603 | 0.88588336 | -0.015997871 | 0.063435923 | 0.800894633 | 7.014718182 | 12 | 0.856639381 | 7.045016523 | 13 | 0.899816784 | -0.002827712 | 0.016245223 | 0.864716999 |
| 38 | Gut microbiota abundance (family Peptococcaceae id.2024) | 9 | MR Egger Weighted median Inverse variance weighted | -0.033926454 | 0.246973398 | 0.894606973 | -0.1371909 | 0.112477881 | 0.222573129 | -0.130827244 | 0.088293244 | 0.138410659 | 2.945046772 | 7 | 0.890038871 | 3.121545539 | 8 | 0.926499786 | -0.00935476 | 0.022267005 | 0.686994799 |
| 39 | Gut microbiota abundance (family Peptostreptococcaceae id.2042) | 13 | MR Egger Weighted median Inverse variance weighted | -0.397061157 | 0.203069522 | 0.076424783 | -0.16378637 | 0.116004037 | 0.157978721 | -0.111665752 | 0.094407464 | 0.236885884 | 12.65339201 | 11 | 0.316581713 | 15.46838555 | 12 | 0.216815311 | 0.025096253 | 0.016042695 | 0.146031953 |
| 40 | Gut microbiota abundance (family Porphyromonadaceae id.943) | 9 | MR Egger Weighted median Inverse variance weighted | -0.295043826 | 0.662304696 | 0.669423799 | 0.113697001 | 0.181692801 | 0.531469074 | -0.017219535 | 0.142137601 | 0.903574651 | 9.070744991 | 7 | 0.247616689 | 9.311087735 | 8 | 0.316736458 | 0.016670837 | 0.038709214 | 0.679659288 |
| 41 | Gut microbiota abundance (family Prevotellaceae id.960) | 16 | MR Egger Weighted median Inverse variance weighted | 0.547567406 | 0.37187696 | 0.163024239 | 0.036876288 | 0.125913054 | 0.769620722 | 0.070427291 | 0.104428631 | 0.500053253 | 21.31509143 | 14 | 0.093826237 | 24.02461156 | 15 | 0.064676717 | -0.03422885 | 0.025658172 | 0.203488799 |
| 42 | Gut microbiota abundance (family Rhodospirillaceae id.2717) | 15 | MR Egger Weighted median Inverse variance weighted | 0.2095692 | 0.271172185 | 0.453447602 | 0.012345439 | 0.083294987 | 0.882174298 | -0.022977126 | 0.0638517 | 0.718958234 | 5.430160403 | 13 | 0.964464023 | 6.208736344 | 14 | 0.960952736 | -0.023706907 | 0.026867323 | 0.393600595 |
| 43 | Gut microbiota abundance (family Rikenellaceae id.967) | 17 | Weighted median Inverse variance weighted | -1.041061442 | 0.313969025 | 0.004703914 | 0.014209604 | 0.128473487 | 0.911930855 | -0.038379356 | 0.129734573 | 0.767360047 | 20.61157153 | 15 | 0.149705474 | 36.24505586 | 16 | 0.002675076 | 0.075443135 | 0.022366692 | 0.004183117 |
| 44 | Gut microbiota abundance (family Ruminococcaceae id.2050) | 9 | MR Egger Weighted median Inverse variance weighted | 0.304427502 | 0.245452438 | 0.254833102 | 0.089278697 | 0.143853618 | 0.534848465 | 0.103401922 | 0.107630967 | 0.336699048 | 7.334339894 | 7 | 0.394917891 | 8.20883854 | 8 | 0.413340755 | -0.021327576 | 0.023344976 | 0.391316307 |
| 45 | Gut microbiota abundance (family Streptococcaceae id.1850) | 13 | MR Egger Weighted median Inverse variance weighted | -0.091194449 | 0.434865234 | 0.837729503 | -0.226323173 | 0.141222802 | 0.10902376 | -0.157957603 | 0.103763186 | 0.127936572 | 13.34838995 | 11 | 0.27116589 | 13.37888177 | 12 | 0.342113743 | -0.005216637 | 0.032909176 | 0.876922796 |
| 46 | Gut microbiota abundance (unknown family id.1000001214) | 9 | MR Egger Weighted median Inverse variance weighted | -0.280060621 | 0.242574508 | 0.286168786 | 0.014247317 | 0.100335084 | 0.887082095 | -0.061248514 | 0.080803381 | 0.448454309 | 9.097365919 | 7 | 0.245740986 | 10.28839264 | 8 | 0.245365145 | 0.026424709 | 0.027603126 | 0.370298089 |
| 47 | Gut microbiota abundance (unknown family id.1000005471) | 13 | MR Egger Weighted median Inverse variance weighted | -0.13646416 | 0.243128883 | 0.585857221 | -0.06649328 | 0.099438448 | 0.503694637 | -0.026797878 | 0.077043056 | 0.727967939 | 9.11798007 | 11 | 0.611003095 | 9.344147678 | 12 | 0.673288414 | 0.009440737 | 0.01985138 | 0.643684175 |
| 48 | Gut microbiota abundance (unknown family id.1000006161) | 13 | MR Egger | -0.138695705 | 0.228815798 | 0.556719493 | -0.161834963 | 0.072223319 | 0.025041771 | -0.125014876 | 0.053728312 | 0.019976072 | 10.31062748 | 11 | 0.502681441 | 10.31441089 | 12 | 0.588398585 | 0.001620207 | 0.026340789 | 0.952056881 |
| 49 | Gut microbiota abundance (family Veillonellaceae id.2172) | 19 | MR Egger Weighted median Inverse variance weighted | -0.002468646 | 0.182151178 | 0.989344598 | 0.049115777 | 0.113341412 | 0.664765155 | -0.054814913 | 0.087673687 | 0.531829894 | 27.45255148 | 17 | 0.051755775 | 27.62889807 | 18 | 0.067931742 | -0.004668148 | 0.014126285 | 0.745091469 |
| 50 | Gut microbiota abundance (family Verrucomicrobiaceae id.4036) | 11 | MR Egger Weighted median Inverse variance weighted | -0.285108179 | 0.294754924 | 0.358680307 | 0.097579872 | 0.119765955 | 0.415212747 | 0.054395955 | 0.088162039 | 0.537234897 | 5.706774301 | 9 | 0.768868874 | 7.163809912 | 10 | 0.709895092 | 0.027977944 | 0.023178254 | 0.258168779 |
| 51 | Gut microbiota abundance (family Victivallaceae id.2255) | 12 | MR Egger Weighted median Inverse variance weighted | 0.018844913 | 0.352642851 | 0.958434566 | -0.006936789 | 0.072010312 | 0.923258094 | 0.060112523 | 0.070684633 | 0.395084567 | 23.63130447 | 10 | 0.008641526 | 23.66516066 | 11 | 0.014221145 | 0.006063666 | 0.050659405 | 0.907095497 |
| 52 | Gut microbiota abundance (genus Actinomyces id.423) | 7 | MR Egger Weighted median Inverse variance weighted | -0.160620031 | 0.209005792 | 0.476902257 | -0.11238795 | 0.116572299 | 0.334993231 | -0.024061609 | 0.085649639 | 0.778763584 | 3.868304902 | 5 | 0.568528511 | 4.381357285 | 6 | 0.625214257 | 0.016887495 | 0.023576774 | 0.505871976 |
| 53 | Gut microbiota abundance (genus Adlercreutzia id.812) | 8 | MR Egger Weighted median Inverse variance weighted | 0.287076791 | 0.411345871 | 0.51136775 | 0.116195193 | 0.116407767 | 0.318195046 | 0.062618736 | 0.09174793 | 0.494917511 | 2.940572712 | 6 | 0.816267798 | 3.253913875 | 7 | 0.860561943 | -0.020318491 | 0.036298003 | 0.595900172 |
| 54 | Gut microbiota abundance (genus Akkermansia id.4037) | 11 | MR Egger Weighted median Inverse variance weighted | -0.282333767 | 0.294601982 | 0.362918284 | 0.097531326 | 0.120088662 | 0.416699264 | 0.054606271 | 0.08813093 | 0.535518577 | 5.723941924 | 9 | 0.767197752 | 7.160589558 | 10 | 0.710202331 | 0.027776896 | 0.023174404 | 0.261290034 |
| 55 | Gut microbiota abundance (genus Alistipes id.968) | 13 | MR Egger Weighted median Inverse variance weighted | -0.624177667 | 0.589463605 | 0.312347962 | 0.042809535 | 0.150110965 | 0.775501699 | 0.040477943 | 0.124084484 | 0.744263381 | 13.65114661 | 11 | 0.252901379 | 15.30008 | 12 | 0.225434722 | 0.039909359 | 0.034622738 | 0.273471082 |
| 56 | Gut microbiota abundance (genus Allisonella id.2174) | 8 | MR Egger Weighted median Inverse variance weighted | -0.512746798 | 0.45521755 | 0.303030162 | 0.054734329 | 0.08039076 | 0.495964204 | 0.039133335 | 0.069654726 | 0.574239473 | 9.288407595 | 6 | 0.15799608 | 11.61453663 | 7 | 0.113969058 | 0.07927755 | 0.064673792 | 0.266197809 |
| 57 | Gut microbiota abundance (genus Alloprevotella id.961) | 6 | MR Egger Weighted median Inverse variance weighted | 0.310190653 | 0.583859503 | 0.623359362 | -0.073365588 | 0.074954836 | 0.327680167 | -0.087492261 | 0.062726589 | 0.163070317 | 0.952831351 | 4 | 0.916861948 | 1.422183706 | 5 | 0.92187132 | -0.055746269 | 0.081370372 | 0.530923783 |
| 58 | Gut microbiota abundance (genus Anaerofilum id.2053) | 11 | MR Egger Weighted median Inverse variance weighted | -0.047434344 | 0.331174305 | 0.889263506 | -0.079242141 | 0.078408066 | 0.312189905 | -0.07280343 | 0.060867515 | 0.231658852 | 3.978234412 | 9 | 0.91283923 | 3.984307649 | 10 | 0.948052115 | -0.002856505 | 0.036654293 | 0.939588057 |
| 59 | Gut microbiota abundance (genus Anaerostipes id.1991) | 13 | MR Egger Weighted median Inverse variance weighted | 0.352163522 | 0.527825285 | 0.518397508 | -0.07456058 | 0.145339666 | 0.607944913 | 0.144847886 | 0.139804869 | 0.300168633 | 21.81825108 | 11 | 0.025819692 | 22.14919353 | 12 | 0.035881335 | -0.013681706 | 0.033494818 | 0.690765913 |
| 60 | Gut microbiota abundance (genus Anaerotruncus id.2054) | 13 | MR Egger Weighted median Inverse variance weighted | 0.456867705 | 0.286841558 | 0.139522712 | 0.005002688 | 0.138758032 | 0.971239843 | 0.014001462 | 0.105048319 | 0.893967258 | 11.03228768 | 11 | 0.440562292 | 13.74125484 | 12 | 0.317535605 | -0.0320041 | 0.019473329 | 0.128531566 |
| 61 | Gut microbiota abundance (genus Bacteroides id.918) | 9 | MR Egger Weighted median Inverse variance weighted | 0.052504943 | 0.814910127 | 0.950428927 | -0.081456836 | 0.167630644 | 0.627016472 | -0.111757951 | 0.147430035 | 0.448426684 | 12.45158302 | 7 | 0.086652543 | 12.52664878 | 8 | 0.129207339 | -0.0108572 | 0.052851844 | 0.843084703 |
| 62 | Gut microbiota abundance (genus Barnesiella id.944) | 13 | MR Egger Weighted median Inverse variance weighted | -0.015510203 | 0.37333865 | 0.967606231 | -0.041607248 | 0.117642472 | 0.723582163 | 0.009135214 | 0.091892996 | 0.920811612 | 5.809894249 | 11 | 0.885742613 | 5.81453308 | 12 | 0.925140989 | 0.001920801 | 0.028201878 | 0.946921169 |
| 63 | Gut microbiota abundance (genus Bifidobacterium id.436) | 13 | MR Egger Weighted median Inverse variance weighted | 0.045670412 | 0.195201985 | 0.819309612 | 0.042732158 | 0.107832615 | 0.691896777 | 0.010004423 | 0.078359326 | 0.898407229 | 10.78843556 | 11 | 0.461154057 | 10.82823275 | 12 | 0.543693782 | -0.003122669 | 0.015653077 | 0.845517467 |
| 64 | Gut microbiota abundance (genus Bilophila id.3170) | 13 | MR Egger Weighted median Inverse variance weighted | 0.023451449 | 0.449869117 | 0.959360142 | 0.017317871 | 0.120314336 | 0.885548996 | 0.083672327 | 0.0914519 | 0.360226999 | 6.023932123 | 11 | 0.871762663 | 6.042623892 | 12 | 0.913918167 | 0.004456695 | 0.032597758 | 0.893724 |
| 65 | Gut microbiota abundance (genus Blautia id.1992) | 13 | MR Egger Weighted median Inverse variance weighted | 0.023451449 | 0.449869117 | 0.959360142 | 0.017317871 | 0.120314336 | 0.885548996 | 0.083672327 | 0.0914519 | 0.360226999 | 6.023932123 | 11 | 0.871762663 | 6.042623892 | 12 | 0.913918167 | 0.004456695 | 0.032597758 | 0.893724 |
| 66 | Gut microbiota abundance (genus Butyricicoccus id.2055) | 8 | MR Egger Weighted median Inverse variance weighted | -0.008084 | 0.221941561 | 0.972125872 | 0.058859907 | 0.153257258 | 0.700934342 | -0.044683798 | 0.112715548 | 0.691788013 | 3.906255898 | 6 | 0.689360951 | 3.942902292 | 7 | 0.786329991 | -0.003625668 | 0.018939671 | 0.854500655 |
| 67 | Gut microbiota abundance (genus Butyricimonas id.945) | 13 | Weighted median Inverse variance weighted | -0.717211084 | 0.302878828 | 0.037284667 | -0.053986798 | 0.120236887 | 0.653429044 | -0.112632703 | 0.085518773 | 0.187821067 | 7.818998635 | 11 | 0.729420109 | 12.14416248 | 12 | 0.434172366 | 0.053795312 | 0.025866826 | 0.061729814 |
| 68 | Gut microbiota abundance (genus Butyrivibrio id.1993) | 15 | MR Egger Weighted median Inverse variance weighted | -0.258523833 | 0.217958353 | 0.256802944 | -0.018478937 | 0.060796523 | 0.761168073 | -0.010874663 | 0.051231384 | 0.831899787 | 19.5318412 | 13 | 0.107514262 | 21.58189513 | 14 | 0.087623338 | 0.036358012 | 0.031125576 | 0.263740167 |
| 69 | Gut microbiota abundance (genus Candidatus Soleaferrea id.11350) | 11 | MR Egger Weighted median Inverse variance weighted | 0.045329611 | 0.341178606 | 0.897225826 | 0.053122726 | 0.085283965 | 0.533355287 | 0.015580507 | 0.065274659 | 0.811344805 | 5.410561592 | 9 | 0.797151483 | 5.41845345 | 10 | 0.861531738 | -0.002949639 | 0.033203149 | 0.93115756 |
| 70 | Gut microbiota abundance (genus Catenibacterium id.2153) | 4 | MR Egger Weighted median Inverse variance weighted | 1.100066071 | 1.094120531 | 0.420563828 | 0.026259811 | 0.096440845 | 0.785399578 | 0.024430976 | 0.086866898 | 0.778521652 | 2.280769693 | 2 | 0.319695964 | 3.39000214 | 3 | 0.335311178 | -0.138640458 | 0.140573811 | 0.427979891 |
| 71 | Gut microbiota abundance (genus Christensenellaceae R 7group id.11283) | 10 | MR Egger Weighted median Inverse variance weighted | 0.567487649 | 0.431382061 | 0.224787051 | 0.039693233 | 0.168566278 | 0.813839674 | 0.110140257 | 0.140954338 | 0.434573371 | 11.66668506 | 8 | 0.166706578 | 13.49578193 | 9 | 0.141425547 | -0.035394092 | 0.031603935 | 0.295238429 |
| 72 | Gut microbiota abundance (genus Clostridium innocuum group id.14397) | 9 | MR Egger Weighted median Inverse variance weighted | -0.120161456 | 0.279235477 | 0.679898856 | -0.005902484 | 0.072105702 | 0.93475902 | -0.022969462 | 0.057787979 | 0.691014854 | 1.878110343 | 7 | 0.966277455 | 2.004680138 | 8 | 0.98086803 | 0.012974448 | 0.036468999 | 0.732495433 |
| 73 | Gut microbiota abundance (genus Clostridium sensustricto1 id.1873) | 7 | MR Egger Weighted median Inverse variance weighted | 0.182332206 | 0.33137775 | 0.605841774 | 0.075292164 | 0.13448268 | 0.57557143 | 0.062128588 | 0.119734254 | 0.60383931 | 7.506823595 | 5 | 0.185591964 | 7.739768354 | 6 | 0.257795207 | -0.013016912 | 0.033046434 | 0.709894323 |
| 74 | Gut microbiota abundance (genus Collinsella id.815) | 9 | MR Egger Weighted median Inverse variance weighted | -0.027495313 | 0.44492004 | 0.952450933 | -0.008093621 | 0.146845903 | 0.956045711 | 0.052311653 | 0.119098983 | 0.660496063 | 3.70362894 | 7 | 0.813210352 | 3.738287355 | 8 | 0.879930547 | 0.005804726 | 0.03118009 | 0.857593383 |
| 75 | Gut microbiota abundance (genus Coprobacter id.949) | 10 | MR Egger Weighted median Inverse variance weighted | -0.157753546 | 0.269190312 | 0.574012912 | 0.015907819 | 0.089245909 | 0.858528966 | 0.015748451 | 0.071890313 | 0.82660159 | 3.469576771 | 8 | 0.901537926 | 3.91690347 | 9 | 0.916796043 | 0.019006977 | 0.028418466 | 0.522434772 |
| 76 | Gut microbiota abundance (genus Coprococcus1 id.11301) | 11 | MR Egger Weighted median Inverse variance weighted | 0.4969865 | 0.254270141 | 0.08236111 | 0.129813603 | 0.141115455 | 0.357619479 | 0.136886453 | 0.109930086 | 0.213053193 | 9.812382108 | 9 | 0.365886513 | 12.42796018 | 10 | 0.257434914 | -0.028533437 | 0.018421976 | 0.155819149 |
| 77 | Gut microbiota abundance (genus Coprococcus2 id.11302) | 8 | MR Egger Weighted median | 0.636192891 | 0.850687116 | 0.482817228 | -0.235401336 | 0.141436495 | 0.09604038 | -0.23510373 | 0.10747457 | 0.028703935 | 5.297759691 | 6 | 0.506228919 | 6.363816202 | 7 | 0.497967462 | -0.062909957 | 0.060929733 | 0.341648307 |
| 78 | Gut microbiota abundance (genus Coprococcus3 id.11303) | 9 | MR Egger Weighted median Inverse variance weighted | -0.196023509 | 0.927634918 | 0.838661594 | 0.044132838 | 0.188094775 | 0.814495259 | -0.06042796 | 0.155250399 | 0.69710674 | 12.91572015 | 7 | 0.074188674 | 12.95644334 | 8 | 0.113357071 | 0.008147506 | 0.054842095 | 0.886087673 |
| 79 | Gut microbiota abundance (genus Defluviitaleaceae UCG011 id.11287) | 9 | MR Egger Weighted median Inverse variance weighted | -0.690522669 | 0.398195727 | 0.126486759 | -0.001897436 | 0.125278952 | 0.987915951 | -0.059680051 | 0.121167185 | 0.622335521 | 10.99288381 | 7 | 0.138929727 | 15.25965556 | 8 | 0.054288964 | 0.065555354 | 0.039770886 | 0.14327605 |
| 80 | Gut microbiota abundance (genus Desulfovibrio id.3173) | 10 | Weighted median Inverse variance weighted | 0.754557639 | 0.262392519 | 0.020647945 | 0.072867711 | 0.119186667 | 0.540951559 | 0.014364565 | 0.10979296 | 0.895907252 | 6.657671571 | 8 | 0.573976618 | 15.51494067 | 9 | 0.077728242 | -0.078339239 | 0.026322639 | 0.017705042 |
| 81 | Gut microbiota abundance (genus Dialister id.2183) | 11 | MR Egger Weighted median Inverse variance weighted | -0.089174621 | 0.560775571 | 0.877164259 | 0.089320049 | 0.124100511 | 0.471685356 | -0.050994739 | 0.129274435 | 0.693234913 | 19.71018462 | 9 | 0.019787663 | 19.72097301 | 10 | 0.032005053 | 0.002892272 | 0.041208309 | 0.945579929 |
| 82 | Gut microbiota abundance (genus Dorea id.1997) | 10 | MR Egger Weighted median Inverse variance weighted | -0.226018263 | 0.326454702 | 0.508316493 | -0.099432502 | 0.162862602 | 0.541510785 | 0.016933923 | 0.11763302 | 0.885535591 | 6.428818267 | 8 | 0.599313497 | 7.065316593 | 9 | 0.630319002 | 0.017192281 | 0.021549385 | 0.44801323 |
| 83 | Gut microbiota abundance (genus Eggerthella id.819) | 10 | MR Egger Weighted median Inverse variance weighted | 0.077280044 | 0.301007109 | 0.803860193 | 0.016530219 | 0.082609822 | 0.841402434 | 0.02210162 | 0.065176927 | 0.734532965 | 5.23244154 | 8 | 0.732468119 | 5.267698089 | 9 | 0.810375854 | -0.006202264 | 0.033031655 | 0.85573421 |
| 84 | Gut microbiota abundance (genus Eisenbergiella id.11304) | 11 | MR Egger Weighted median Inverse variance weighted | 0.51559838 | 0.463775415 | 0.29506376 | -0.031945512 | 0.081965315 | 0.696725656 | 0.011676998 | 0.062783508 | 0.852453938 | 5.382381279 | 9 | 0.799783089 | 6.585041618 | 10 | 0.763952389 | -0.053139095 | 0.048455453 | 0.301265307 |
| 85 | Gut microbiota abundance (genus Enterorhabdus id.820) | 6 | MR Egger Weighted median Inverse variance weighted | -0.068082364 | 0.26397176 | 0.809198232 | 0.064668783 | 0.124098349 | 0.602290753 | 0.084714853 | 0.100140223 | 0.39757378 | 2.735603747 | 4 | 0.602998947 | 3.126983548 | 5 | 0.680416055 | 0.02141416 | 0.034229602 | 0.565497449 |
| 86 | Gut microbiota abundance (genus Erysipelatoclostridium id.11381) | 15 | MR Egger Weighted median Inverse variance weighted | -0.002111279 | 0.322676643 | 0.994878807 | 0.012520104 | 0.096872427 | 0.897165204 | 0.002224225 | 0.079484244 | 0.977675539 | 18.259156 | 13 | 0.147925059 | 18.25942729 | 14 | 0.195219341 | 0.000360009 | 0.025904055 | 0.989122541 |
| 87 | Gut microbiota abundance (genus Erysipelotrichaceae UCG003 id.11384) | 15 | MR Egger Weighted median Inverse variance weighted | -0.002111279 | 0.322676643 | 0.994878807 | 0.012520104 | 0.096872427 | 0.897165204 | 0.002224225 | 0.079484244 | 0.977675539 | 18.259156 | 13 | 0.147925059 | 18.25942729 | 14 | 0.195219341 | 0.000360009 | 0.025904055 | 0.989122541 |
| 88 | Gut microbiota abundance (genus Escherichia Shigella id.3504) | 10 | MR Egger Weighted median Inverse variance weighted | -0.174278716 | 0.380465815 | 0.659090206 | 0.173432287 | 0.149194773 | 0.245050485 | 0.042969244 | 0.11783473 | 0.715367555 | 11.55079243 | 8 | 0.1723996 | 12.07574038 | 9 | 0.209073033 | 0.017095537 | 0.02835212 | 0.56322629 |
| 89 | Gut microbiota abundance (genus Eubacterium brachy group id.11296) | 10 | MR Egger Weighted median Inverse variance weighted | -0.001651871 | 0.227938477 | 0.994395237 | 0.05061437 | 0.075228517 | 0.50106926 | 0.051775206 | 0.056810082 | 0.362098587 | 5.636864278 | 8 | 0.687833969 | 5.695442909 | 9 | 0.769970113 | 0.006926183 | 0.028617017 | 0.814845687 |
| 90 | Gut microbiota abundance (genus Eubacterium coprostanoligenes group id.11375) | 13 | MR Egger Weighted median Inverse variance weighted | -0.544583259 | 0.41300813 | 0.214104227 | -0.097409371 | 0.142799364 | 0.495149486 | -0.072512486 | 0.104941065 | 0.489576257 | 8.049676748 | 11 | 0.708847059 | 9.446308117 | 12 | 0.664410728 | 0.030019769 | 0.025401915 | 0.262206785 |
| 91 | Gut microbiota abundance (genus Eubacterium eligens group id.14372) | 6 | MR Egger Weighted median Inverse variance weighted | -0.330528249 | 0.633615452 | 0.629462858 | -0.051739945 | 0.179866062 | 0.7736084 | -0.124148053 | 0.153975581 | 0.420079095 | 6.279510495 | 4 | 0.17922405 | 6.458939839 | 5 | 0.264086392 | 0.016881881 | 0.049935224 | 0.752305096 |
| 92 | Gut microbiota abundance (genus Eubacterium fissicatena group id.14373) | 9 | MR Egger Weighted median Inverse variance weighted | 0.04873437 | 0.338111466 | 0.889454218 | -0.072709804 | 0.08703214 | 0.403472133 | -0.0431555 | 0.061396761 | 0.48212095 | 8.146350061 | 7 | 0.31986608 | 8.2356351 | 8 | 0.410797784 | -0.012096931 | 0.043673538 | 0.789792232 |
| 93 | Gut microbiota abundance (genus Eubacterium hallii group id.11338) | 16 | MR Egger Weighted median Inverse variance weighted | -0.192428983 | 0.165050509 | 0.263141951 | 0.055240664 | 0.112759164 | 0.624204979 | 0.048472771 | 0.084597882 | 0.566659225 | 15.03835691 | 14 | 0.375537689 | 18.02882035 | 15 | 0.26115342 | 0.022541036 | 0.013509547 | 0.117412474 |
| 94 | Gut microbiota abundance (genus Eubacterium nodatum group id.11297) | 11 | MR Egger Weighted median Inverse variance weighted | 0.070168207 | 0.220348541 | 0.757415323 | -0.079859866 | 0.064298029 | 0.214226728 | -0.081164194 | 0.049470543 | 0.10086863 | 4.371892408 | 9 | 0.885276943 | 4.868604842 | 10 | 0.899780622 | -0.02255163 | 0.031998191 | 0.498779239 |
| 95 | Gut microbiota abundance (genus Eubacterium oxidoreducens group id.11339) | 5 | MR Egger Weighted median Inverse variance weighted | 0.003741877 | 0.338720008 | 0.991879433 | -0.098016629 | 0.113461786 | 0.387657392 | -0.134241589 | 0.090713875 | 0.13891722 | 0.523987235 | 3 | 0.913590787 | 0.702757536 | 4 | 0.950988426 | -0.01537981 | 0.036375024 | 0.700909273 |
| 96 | Gut microbiota abundance (genus Eubacterium rectale group id.14374) | 8 | MR Egger Weighted median Inverse variance weighted | 0.262907873 | 0.457610755 | 0.58649304 | 0.060386913 | 0.152689326 | 0.69248221 | 0.098336387 | 0.125081229 | 0.43176192 | 0.47173334 | 6 | 0.998165656 | 0.611511694 | 7 | 0.998927155 | -0.010838615 | 0.028990374 | 0.721356687 |
| 97 | Gut microbiota abundance (genus Eubacterium ruminantium group id.11340) | 18 | Weighted median Inverse variance weighted | -0.398303611 | 0.183532717 | 0.045389282 | -0.106962588 | 0.077499322 | 0.16753292 | -0.030681818 | 0.060173242 | 0.610127562 | 16.1361733 | 16 | 0.443497953 | 20.58042791 | 17 | 0.245621928 | 0.037021569 | 0.017635819 | 0.052017412 |
| 98 | Gut microbiota abundance (genus Eubacterium ventriosum group id.11341) | 15 | MR Egger Weighted median Inverse variance weighted | 0.226157208 | 0.403742681 | 0.584899883 | -0.050630875 | 0.118830012 | 0.670050879 | 0.02517174 | 0.087835772 | 0.774435983 | 13.73141785 | 13 | 0.393027144 | 14.00693822 | 14 | 0.449194277 | -0.015419322 | 0.030190788 | 0.618102685 |
| 99 | Gut microbiota abundance (genus Eubacterium xylanophilum group id.14375) | 9 | MR Egger Weighted median Inverse variance weighted | -0.642123276 | 0.410619169 | 0.161842196 | -0.070032041 | 0.144337077 | 0.627536 | -0.088549726 | 0.146327277 | 0.545080357 | 14.10143956 | 7 | 0.049406287 | 18.22589131 | 8 | 0.01959526 | 0.048117358 | 0.033628025 | 0.195555389 |
| 100 | Gut microbiota abundance (genus Faecalibacterium id.2057) | 10 | MR Egger Weighted median Inverse variance weighted | -0.079567756 | 0.217204517 | 0.723620227 | 0.053843308 | 0.125750129 | 0.668522292 | 0.088331118 | 0.110380956 | 0.423572483 | 12.53182849 | 8 | 0.129005447 | 13.80320219 | 9 | 0.129499934 | 0.020376742 | 0.022618321 | 0.393955698 |
| 101 | Gut microbiota abundance (genus Family XIII AD3011 group id.11293) | 13 | MR Egger Weighted median Inverse variance weighted | -0.121758848 | 0.45159084 | 0.792441856 | 0.000796908 | 0.129252493 | 0.995080663 | -0.036729217 | 0.092023176 | 0.689797364 | 11.86546315 | 11 | 0.373840488 | 11.90549855 | 12 | 0.453298768 | 0.007018979 | 0.036433244 | 0.850741382 |
| 102 | Gut microbiota abundance (genus Family XIII UCG001 id.11294) | 8 | MR Egger Weighted median Inverse variance weighted | 0.470605815 | 0.430851003 | 0.316619547 | 0.000457896 | 0.145795868 | 0.997494118 | 0.108845519 | 0.137887562 | 0.429890639 | 10.31825176 | 6 | 0.111873804 | 11.67381315 | 7 | 0.111808943 | -0.032169627 | 0.036233798 | 0.408806934 |
| 103 | Gut microbiota abundance (genus Flavonifractor id.2059) | 5 | MR Egger Weighted median Inverse variance weighted | 0.196927816 | 0.501723458 | 0.720911862 | 0.152061058 | 0.165025158 | 0.356819878 | 0.152769782 | 0.125598744 | 0.223858444 | 2.117433997 | 3 | 0.548393807 | 2.125698123 | 4 | 0.712652757 | -0.003738406 | 0.041123299 | 0.933296038 |
| 104 | Gut microbiota abundance (genus Fusicatenibacter id.11305) | 18 | MR Egger Weighted median Inverse variance weighted | -0.218120124 | 0.349127874 | 0.540938315 | 0.007880233 | 0.128850977 | 0.951233593 | 0.026091661 | 0.091623262 | 0.775819482 | 18.21639079 | 16 | 0.311378743 | 18.81593095 | 17 | 0.339199326 | 0.01706452 | 0.023515603 | 0.478523222 |
| 105 | Gut microbiota abundance (genus Gordonibacter id.821) | 12 | MR Egger Weighted median Inverse variance weighted | 0.170714331 | 0.270988659 | 0.542840692 | -0.103899801 | 0.072127409 | 0.149724941 | -0.060452526 | 0.06241241 | 0.332745874 | 15.90392567 | 10 | 0.102415865 | 17.12754099 | 11 | 0.10415915 | -0.035750091 | 0.040757438 | 0.400990521 |
| 106 | Gut microbiota abundance (genus Haemophilus id.3698) | 9 | MR Egger Weighted median Inverse variance weighted | 0.05617964 | 0.211486027 | 0.798168216 | -0.030699027 | 0.10082978 | 0.760774495 | -0.085734395 | 0.091983659 | 0.351304925 | 10.79035949 | 7 | 0.148028449 | 11.6581047 | 8 | 0.167122623 | -0.018644622 | 0.024850001 | 0.477537953 |
| 107 | Gut microbiota abundance (genus Holdemanella id.11393) | 11 | MR Egger Weighted median Inverse variance weighted | 0.233084096 | 0.188626733 | 0.247854688 | 0.117965208 | 0.085323342 | 0.166797776 | 0.113636152 | 0.065802213 | 0.084179318 | 3.779725687 | 9 | 0.92530306 | 4.236293995 | 10 | 0.936058366 | -0.013885334 | 0.020549603 | 0.516216421 |
| 108 | Gut microbiota abundance (genus Holdemania id.2157) | 14 | Weighted median Inverse variance weighted | 0.457955351 | 0.207332476 | 0.047382677 | -0.017573907 | 0.100597112 | 0.861318556 | -0.041809824 | 0.081743452 | 0.6090177 | 10.92591134 | 12 | 0.535278572 | 17.4949538 | 13 | 0.177653857 | -0.051263582 | 0.020001286 | 0.024863219 |
| 109 | Gut microbiota abundance (genus Howardella id.2000) | 9 | MR Egger Weighted median Inverse variance weighted | 0.205054169 | 0.228325518 | 0.39897931 | -0.002884133 | 0.070725074 | 0.967471682 | -0.014525386 | 0.057388782 | 0.800187095 | 8.125587046 | 7 | 0.321650221 | 9.271679306 | 8 | 0.319898449 | -0.035171308 | 0.035396236 | 0.353493918 |
| 110 | Gut microbiota abundance (genus Hungatella id.11306) | 5 | MR Egger Weighted median Inverse variance weighted | 0.180146378 | 0.491970877 | 0.738535367 | -0.059720089 | 0.104145567 | 0.566354251 | -0.007267262 | 0.081252044 | 0.928731458 | 2.329086475 | 3 | 0.506971623 | 2.47827459 | 4 | 0.648530244 | -0.024617959 | 0.063736019 | 0.725077474 |
| 111 | Gut microbiota abundance (genus Intestinibacter id.11345) | 15 | MR Egger Weighted median Inverse variance weighted | -0.114924837 | 0.251547995 | 0.655304641 | -0.045998082 | 0.106255371 | 0.665086515 | -0.002037767 | 0.077207656 | 0.978943616 | 9.484668922 | 13 | 0.735433513 | 9.707008763 | 14 | 0.783275455 | 0.009460132 | 0.020062662 | 0.645078411 |
| 112 | Gut microbiota abundance (genus Intestinimonas id.2062) | 16 | MR Egger Weighted median Inverse variance weighted | -0.067109429 | 0.204782557 | 0.747979672 | -0.043688729 | 0.103803879 | 0.673844448 | -0.016833476 | 0.073859228 | 0.819713901 | 10.06203819 | 14 | 0.75763384 | 10.13132608 | 15 | 0.8113988 | 0.004626943 | 0.017577837 | 0.796210899 |
| 113 | Gut microbiota abundance (genus Lachnoclostridium id.11308) | 13 | MR Egger Weighted median | 0.370924264 | 0.339284817 | 0.297646112 | 0.207171188 | 0.134169701 | 0.122564555 | 0.212364444 | 0.099729267 | 0.033220398 | 8.766184329 | 11 | 0.643464574 | 9.005241402 | 12 | 0.702482684 | -0.011108838 | 0.022720498 | 0.634490422 |
| 114 | Gut microbiota abundance (genus Lachnospiraceae FCS020 group id.11314) | 12 | MR Egger Weighted median Inverse variance weighted | 0.248288352 | 0.237426881 | 0.320297173 | 0.085916392 | 0.117976327 | 0.466459879 | 0.113720821 | 0.089026048 | 0.201465236 | 5.018957253 | 10 | 0.889908061 | 5.392743796 | 11 | 0.910665867 | -0.010679516 | 0.017467861 | 0.554590545 |
| 115 | Gut microbiota abundance (genus Lachnospiraceae NC2004 group id.11316) | 9 | MR Egger Weighted median Inverse variance weighted | -0.296244869 | 0.297056513 | 0.351851565 | -0.108042331 | 0.095976438 | 0.260285221 | -0.084619898 | 0.073067727 | 0.246822319 | 6.274735999 | 7 | 0.508061096 | 6.814942767 | 8 | 0.556724404 | 0.025131896 | 0.034193633 | 0.486235549 |
| 116 | Gut microbiota abundance (genus Lachnospiraceae ND3007 group id.11317) | 3 | MR Egger Weighted median Inverse variance weighted | 3.260940945 | 3.505991933 | 0.523043847 | -0.031720767 | 0.249065599 | 0.898656198 | 0.032404986 | 0.20777759 | 0.876064577 | 0.023692768 | 1 | 0.877669225 | 0.874669003 | 2 | 0.645755389 | -0.190077257 | 0.206049441 | 0.525655183 |
| 117 | Gut microbiota abundance (genus Lachnospiraceae NK4A136 group id.11319) | 15 | MR Egger Weighted median Inverse variance weighted | -0.198152461 | 0.156622433 | 0.228021806 | 0.063809018 | 0.117791138 | 0.588016073 | -0.039766718 | 0.078549739 | 0.612673004 | 12.3882908 | 13 | 0.496089756 | 13.75459396 | 14 | 0.468149588 | 0.013622617 | 0.011654322 | 0.263435776 |
| 118 | Gut microbiota abundance (genus Lachnospiraceae UCG001 id.11321) | 13 | MR Egger Weighted median | 0.25978421 | 0.317399772 | 0.430459526 | 0.170760158 | 0.099028692 | 0.084644618 | 0.157742801 | 0.073254526 | 0.031291003 | 7.037359831 | 11 | 0.796050002 | 7.14653202 | 12 | 0.847780036 | -0.009550629 | 0.028905198 | 0.747296161 |
| 119 | Gut microbiota abundance (genus Lachnospiraceae UCG004 id.11324) | 12 | MR Egger Weighted median Inverse variance weighted | 0.280058336 | 0.471454746 | 0.565687032 | -0.184432662 | 0.140864991 | 0.190437246 | -0.097848411 | 0.111448984 | 0.379962207 | 13.03175 | 10 | 0.221902055 | 13.92019854 | 11 | 0.237440725 | -0.024982076 | 0.030256157 | 0.428241069 |
| 120 | Gut microbiota abundance (genus Lachnospiraceae UCG008 id.11328) | 11 | MR Egger Weighted median Inverse variance weighted | -0.619183165 | 0.347570946 | 0.108525973 | -0.117508197 | 0.095060505 | 0.216406121 | -0.088197174 | 0.070321049 | 0.209766662 | 8.56023615 | 9 | 0.478816452 | 10.98445925 | 10 | 0.358730055 | 0.055474482 | 0.035629273 | 0.153898394 |
| 121 | Gut microbiota abundance (genus Lachnospiraceae UCG010 id.11330) | 10 | MR Egger Weighted median Inverse variance weighted | -0.004104835 | 0.348514402 | 0.990891083 | 0.021092903 | 0.145014606 | 0.884352712 | 0.020923322 | 0.107750709 | 0.84603284 | 9.71994192 | 8 | 0.285235103 | 9.726962309 | 9 | 0.373038056 | 0.002027003 | 0.026666164 | 0.941274515 |
| 122 | Gut microbiota abundance (genus Lachnospira id.2004) | 10 | MR Egger Weighted median Inverse variance weighted | -0.004104835 | 0.348514402 | 0.990891083 | 0.021092903 | 0.145014606 | 0.884352712 | 0.020923322 | 0.107750709 | 0.84603284 | 9.71994192 | 8 | 0.285235103 | 9.726962309 | 9 | 0.373038056 | 0.002027003 | 0.026666164 | 0.941274515 |
| 123 | Gut microbiota abundance (genus Lactobacillus id.1837) | 9 | MR Egger Weighted median Inverse variance weighted | 0.071743508 | 0.206515685 | 0.738501979 | 0.110926333 | 0.094517585 | 0.240553181 | 0.064742722 | 0.07342139 | 0.377886883 | 9.291737056 | 7 | 0.23238362 | 9.293519965 | 8 | 0.318143306 | -0.000888017 | 0.024230149 | 0.971787917 |
| 124 | Gut microbiota abundance (genus Lactococcus id.1851) | 9 | MR Egger Weighted median Inverse variance weighted | -0.122933768 | 0.287195001 | 0.681476463 | -0.013575763 | 0.080364821 | 0.865854305 | 0.030185923 | 0.061116251 | 0.621369797 | 7.835354198 | 7 | 0.347336 | 8.170148523 | 8 | 0.417028596 | 0.02072156 | 0.037889048 | 0.601437571 |
| 125 | Gut microbiota abundance (genus Marvinbryantia id.2005) | 10 | MR Egger Weighted median Inverse variance weighted | 0.651312136 | 0.388642642 | 0.132292224 | 0.057241237 | 0.124815823 | 0.646517406 | 0.021857524 | 0.099284772 | 0.825754482 | 3.02823276 | 8 | 0.932573303 | 5.834560059 | 9 | 0.756355277 | -0.056020747 | 0.033441037 | 0.132422916 |
| 126 | Gut microbiota abundance (genus Methanobrevibacter id.123) | 6 | MR Egger Weighted median Inverse variance weighted | -0.234773336 | 0.276368092 | 0.443459629 | 0.016676977 | 0.092895961 | 0.857526928 | 0.046551408 | 0.073669571 | 0.527455722 | 1.963377119 | 4 | 0.742494905 | 3.078828148 | 5 | 0.687834156 | 0.041747081 | 0.039527638 | 0.350466147 |
| 127 | Gut microbiota abundance (genus Odoribacter id.952) | 7 | MR Egger Weighted median Inverse variance weighted | 0.10627685 | 0.458022227 | 0.825709207 | 0.111738796 | 0.165188896 | 0.4987674 | -0.012121794 | 0.134708025 | 0.928298592 | 7.129282448 | 5 | 0.211201609 | 7.235416254 | 6 | 0.299623815 | -0.009245561 | 0.033887873 | 0.795894813 |
| 128 | Gut microbiota abundance (genus Olsenella id.822) | 10 | MR Egger Weighted median Inverse variance weighted | 0.092784495 | 0.166305958 | 0.592168436 | -0.005590046 | 0.070163251 | 0.936498137 | -0.027298535 | 0.051897459 | 0.598881902 | 6.160684888 | 8 | 0.62923819 | 6.738306501 | 9 | 0.664344253 | -0.017148838 | 0.022563839 | 0.469049165 |
| 129 | Gut microbiota abundance (genus Oscillibacter id.2063) | 13 | MR Egger Weighted median Inverse variance weighted | -0.054997736 | 0.299543302 | 0.857663775 | 0.031697328 | 0.099636915 | 0.750387573 | 0.02641599 | 0.076071391 | 0.728401537 | 13.78750372 | 11 | 0.244975604 | 13.8870483 | 12 | 0.307979294 | 0.0079328 | 0.028149079 | 0.783317507 |
| 130 | Gut microbiota abundance (genus Oscillospira id.2064) | 8 | MR Egger Weighted median Inverse variance weighted | 0.208895861 | 0.633898383 | 0.752946775 | 0.027806537 | 0.145841144 | 0.848789483 | -0.095399514 | 0.142208483 | 0.502320782 | 14.55337616 | 6 | 0.02402988 | 15.14573533 | 7 | 0.034176011 | -0.030181902 | 0.061074552 | 0.638747284 |
| 131 | Gut microbiota abundance (genus Oxalobacter id.2978) | 11 | Weighted median Inverse variance weighted | -0.617230228 | 0.254475808 | 0.038264132 | 0.136571186 | 0.08108199 | 0.092112292 | 0.102311787 | 0.061792387 | 0.0977756 | 4.610874768 | 9 | 0.866825646 | 12.9861636 | 10 | 0.224446454 | 0.10987005 | 0.037964652 | 0.017767869 |
| 132 | Gut microbiota abundance (genus Parabacteroides id.954) | 5 | MR Egger Weighted median Inverse variance weighted | 0.237151886 | 0.948411321 | 0.818692736 | -0.346807175 | 0.19169369 | 0.070424028 | -0.240365033 | 0.152503695 | 0.114996778 | 2.220219086 | 3 | 0.52797683 | 2.480451306 | 4 | 0.648139677 | -0.041948233 | 0.08223054 | 0.64512301 |
| 133 | Gut microbiota abundance (genus Paraprevotella id.962) | 13 | MR Egger Weighted median Inverse variance weighted | -0.116100149 | 0.23153754 | 0.62595109 | -0.077469621 | 0.085823241 | 0.366703812 | -0.078567033 | 0.060413455 | 0.193433426 | 11.19142154 | 11 | 0.427368816 | 11.22014609 | 12 | 0.51015236 | 0.00413217 | 0.024592227 | 0.869611165 |
| 134 | Gut microbiota abundance (genus Parasutterella id.2892) | 14 | MR Egger Weighted median Inverse variance weighted | 0.02970563 | 0.199036374 | 0.883837464 | 0.087692158 | 0.101421043 | 0.387239295 | 0.04315675 | 0.071905459 | 0.548381354 | 9.286351777 | 12 | 0.678297104 | 9.291604562 | 13 | 0.750580607 | 0.001174031 | 0.016198871 | 0.943417025 |
| 135 | Gut microbiota abundance (genus Peptococcus id.2037) | 12 | MR Egger Weighted median Inverse variance weighted | 0.227619019 | 0.221414032 | 0.328162226 | 0.091785746 | 0.07675697 | 0.231775829 | 0.054684089 | 0.057519381 | 0.34175303 | 10.03470272 | 10 | 0.437453988 | 10.69132213 | 11 | 0.469473882 | -0.023362267 | 0.028880878 | 0.437384638 |
| 136 | Gut microbiota abundance (genus Phascolarctobacterium id.2168) | 8 | MR Egger Weighted median Inverse variance weighted | 0.058937336 | 0.580862187 | 0.922486955 | 0.081419876 | 0.136692471 | 0.551413928 | 0.023588246 | 0.110252745 | 0.830588377 | 8.227324098 | 6 | 0.221917599 | 8.232625055 | 7 | 0.312529037 | -0.00301031 | 0.048415866 | 0.952442081 |
| 137 | Gut microbiota abundance (genus Prevotella7 id.11182) | 11 | Weighted median Inverse variance weighted | -0.755693723 | 0.324253717 | 0.04470589 | -0.087676776 | 0.076439249 | 0.251376447 | -0.040359119 | 0.066288728 | 0.542631598 | 10.69640318 | 9 | 0.297094264 | 16.65838135 | 10 | 0.08227325 | 0.101363824 | 0.045256964 | 0.051865297 |
| 138 | Gut microbiota abundance (genus Prevotella9 id.11183) | 15 | MR Egger Weighted median Inverse variance weighted | -0.092842232 | 0.270062404 | 0.736507917 | -0.040732904 | 0.099194968 | 0.681340357 | -0.046735166 | 0.089300749 | 0.600733678 | 25.39441612 | 13 | 0.020476534 | 25.45893158 | 14 | 0.030297953 | 0.004821234 | 0.026529174 | 0.858595093 |
| 139 | Gut microbiota abundance (genus Rikenellaceae RC9 gut group id.11191) | 11 | MR Egger Weighted median Inverse variance weighted | -0.165012606 | 0.321442222 | 0.620069465 | 0.019311704 | 0.068376841 | 0.777613441 | -0.035502948 | 0.049528515 | 0.473486197 | 9.74968133 | 9 | 0.371127521 | 9.930175056 | 10 | 0.446640521 | 0.018206306 | 0.044603069 | 0.692678098 |
| 140 | Gut microbiota abundance (genus Romboutsia id.11347) | 13 | MR Egger Weighted median Inverse variance weighted | -0.353985098 | 0.259973736 | 0.200554341 | -0.197806723 | 0.11584461 | 0.087725868 | -0.12423115 | 0.089412378 | 0.16470574 | 6.529631774 | 11 | 0.835796745 | 7.415439941 | 12 | 0.828983858 | 0.020021724 | 0.021273141 | 0.366825694 |
| 141 | Gut microbiota abundance (genus Roseburia id.2012) | 13 | MR Egger Weighted median Inverse variance weighted | 0.014308842 | 0.385400392 | 0.97104879 | -0.071200645 | 0.136099121 | 0.600867877 | -0.009252147 | 0.122706646 | 0.939896033 | 17.58659407 | 11 | 0.091682172 | 17.59331187 | 12 | 0.12860848 | -0.001811233 | 0.027941855 | 0.949479151 |
| 142 | Gut microbiota abundance (genus Ruminiclostridium5 id.11355) | 11 | MR Egger Weighted median Inverse variance weighted | 0.138854106 | 0.460168053 | 0.769701443 | 0.03365848 | 0.140651861 | 0.810870269 | 0.081205822 | 0.10932058 | 0.457588959 | 9.143656685 | 9 | 0.424120578 | 9.16057128 | 10 | 0.516935702 | -0.003738366 | 0.028972747 | 0.900171096 |
| 143 | Gut microbiota abundance (genus Ruminiclostridium6 id.11356) | 15 | MR Egger Weighted median Inverse variance weighted | -0.037288271 | 0.231831189 | 0.874690165 | -0.103547614 | 0.124684888 | 0.406270586 | -0.075299644 | 0.090161705 | 0.403626411 | 15.90273487 | 13 | 0.254422052 | 15.94200112 | 14 | 0.316929041 | -0.003581879 | 0.019992425 | 0.860572839 |
| 144 | Gut microbiota abundance (genus Ruminiclostridium9 id.11357) | 8 | MR Egger Weighted median Inverse variance weighted | -1.79738832 | 0.871665137 | 0.084819336 | -0.146216674 | 0.189859394 | 0.441221973 | -0.167230993 | 0.213294256 | 0.433017287 | 11.76546667 | 6 | 0.067410195 | 18.93498453 | 7 | 0.008393466 | 0.106502243 | 0.055698401 | 0.104400028 |
| 145 | Gut microbiota abundance (genus Ruminococcaceae NK4A214 group id.11358) | 13 | MR Egger Weighted median Inverse variance weighted | 0.016732978 | 0.304896226 | 0.957217453 | -0.01089116 | 0.125816186 | 0.931018036 | 0.036755491 | 0.092534337 | 0.69121319 | 7.481121109 | 11 | 0.758891266 | 7.485871172 | 12 | 0.823908324 | 0.001533998 | 0.022257436 | 0.946289653 |
| 146 | Gut microbiota abundance (genus Ruminococcaceae UCG002 id.11360) | 22 | MR Egger Weighted median Inverse variance weighted | 0.014081674 | 0.184511146 | 0.939923746 | -0.044791856 | 0.099213688 | 0.651651913 | -0.103024115 | 0.069890397 | 0.140459752 | 14.8078004 | 20 | 0.787297621 | 15.27809907 | 21 | 0.808743362 | -0.009681329 | 0.014117185 | 0.50072276 |
| 147 | Gut microbiota abundance (genus Ruminococcaceae UCG003 id.11361) | 12 | MR Egger Weighted median Inverse variance weighted | 0.171345837 | 0.294349472 | 0.573376844 | 0.022257859 | 0.119875009 | 0.852699199 | -0.025915788 | 0.08978926 | 0.772865272 | 6.460495484 | 10 | 0.775205508 | 6.955690138 | 11 | 0.802660364 | -0.015308706 | 0.02175457 | 0.497676885 |
| 148 | Gut microbiota abundance (genus Ruminococcaceae UCG004 id.11362) | 11 | MR Egger Weighted median | 0.301629293 | 0.4501786 | 0.519663432 | -0.117035669 | 0.113532559 | 0.302608563 | -0.173621631 | 0.081147472 | 0.032388602 | 8.430214987 | 9 | 0.491438207 | 9.582133644 | 10 | 0.477886057 | -0.040555836 | 0.037787003 | 0.311080618 |
| 149 | Gut microbiota abundance (genus Ruminococcaceae UCG005 id.11363) | 14 | MR Egger Weighted median Inverse variance weighted | 0.13038034 | 0.245791544 | 0.605476788 | 0.113455364 | 0.120362456 | 0.345878255 | 0.104390558 | 0.086984307 | 0.230097421 | 14.17977577 | 12 | 0.289375797 | 14.19505884 | 13 | 0.36025185 | -0.002348491 | 0.020650359 | 0.911335251 |
| 150 | Gut microbiota abundance (genus Ruminococcaceae UCG009 id.11366) | 12 | MR Egger Inverse variance weighted | 0.272684063 | 0.296819442 | 0.379885091 | 0.192027902 | 0.093407241 | 0.03980046 | 0.139208752 | 0.071808567 | 0.052548938 | 12.12627636 | 10 | 0.276694212 | 12.38798312 | 11 | 0.335197284 | -0.013483718 | 0.029024578 | 0.652194935 |
| 151 | Gut microbiota abundance (genus Ruminococcaceae UCG010 id.11367) | 6 | MR Egger | -0.443000115 | 0.3878512 | 0.317101478 | -0.385799371 | 0.161911434 | 0.017182447 | -0.295271855 | 0.129144573 | 0.022232813 | 5.796282049 | 4 | 0.214887371 | 6.038764249 | 5 | 0.302465246 | 0.01127826 | 0.027570644 | 0.703445189 |
| 152 | Gut microbiota abundance (genus Ruminococcaceae UCG011 id.11368) | 8 | MR Egger Weighted median Inverse variance weighted | 0.106470159 | 0.292220924 | 0.728094046 | 0.028782775 | 0.074892829 | 0.700741649 | 0.046379987 | 0.056917965 | 0.415154317 | 6.343699586 | 6 | 0.385809725 | 6.390274791 | 7 | 0.49498774 | -0.008123265 | 0.038703384 | 0.840702791 |
| 153 | Gut microbiota abundance (genus Ruminococcaceae UCG013 id.11370) | 12 | MR Egger Weighted median Inverse variance weighted | -0.065143542 | 0.34841921 | 0.855423883 | -0.038194283 | 0.14672246 | 0.794619616 | 0.065498298 | 0.117796231 | 0.578189996 | 15.61880759 | 10 | 0.111076849 | 15.86940656 | 11 | 0.146048782 | 0.010234199 | 0.025549838 | 0.69716932 |
| 154 | Gut microbiota abundance (genus Ruminococcaceae UCG014 id.11371) | 11 | MR Egger Weighted median Inverse variance weighted | 0.075210633 | 0.210298191 | 0.728857764 | 0.009846668 | 0.135798207 | 0.9421964 | 0.004452877 | 0.089148395 | 0.960162993 | 7.035116012 | 9 | 0.633463189 | 7.173124579 | 10 | 0.709006099 | -0.006710865 | 0.01806448 | 0.718863889 |
| 155 | Gut microbiota abundance (genus Ruminococcus1 id.11373) | 10 | MR Egger Weighted median Inverse variance weighted | 0.212071018 | 0.297123389 | 0.495676087 | 0.060213603 | 0.13836427 | 0.663430522 | 0.064677979 | 0.106972259 | 0.545428954 | 9.734557884 | 8 | 0.284152969 | 10.08305791 | 9 | 0.343803166 | -0.012284315 | 0.022954227 | 0.607086484 |
| 156 | Gut microbiota abundance (genus Ruminococcus2 id.11374) | 15 | MR Egger Weighted median Inverse variance weighted | 0.381543015 | 0.220369543 | 0.107030669 | 0.127095691 | 0.113782139 | 0.263990408 | 0.031925171 | 0.097470997 | 0.74326321 | 17.78564771 | 13 | 0.165829191 | 21.93855602 | 14 | 0.079885725 | -0.030553675 | 0.017536804 | 0.105054015 |
| 157 | Gut microbiota abundance (genus Ruminococcus gauvreauii group id.11342) | 12 | MR Egger Weighted median Inverse variance weighted | -0.133743474 | 0.482314566 | 0.787201362 | 0.038713108 | 0.12312874 | 0.753208422 | 0.048621747 | 0.11171204 | 0.663386936 | 16.5900691 | 10 | 0.083941276 | 16.84188308 | 11 | 0.112640432 | 0.013425201 | 0.034459182 | 0.705002839 |
| 158 | Gut microbiota abundance (genus Ruminococcus gnavus group id.14376) | 12 | MR Egger Weighted median Inverse variance weighted | 0.35015999 | 0.333704707 | 0.318732665 | -0.057474439 | 0.088584576 | 0.516462066 | 0.031427476 | 0.070531983 | 0.655902349 | 12.43948635 | 10 | 0.256719133 | 13.62761038 | 11 | 0.254288254 | -0.034800496 | 0.035608672 | 0.351468549 |
| 159 | Gut microbiota abundance (genus Ruminococcus torques group id.14377) | 9 | MR Egger Weighted median Inverse variance weighted | 0.775258048 | 0.514957271 | 0.175919469 | 0.099368546 | 0.174982771 | 0.570119055 | 0.0018992 | 0.17271761 | 0.991226651 | 10.46400275 | 7 | 0.163767768 | 14.18862747 | 8 | 0.076979473 | -0.04950436 | 0.03136188 | 0.158461998 |
| 160 | Gut microbiota abundance (genus Sellimonas id.14369) | 9 | MR Egger Weighted median Inverse variance weighted | -0.26142607 | 0.372948707 | 0.505947626 | -0.009429023 | 0.072091915 | 0.895940098 | 0.063932898 | 0.063198497 | 0.311719521 | 11.37025597 | 7 | 0.123264241 | 12.64407909 | 8 | 0.124696663 | 0.047688242 | 0.053850885 | 0.405246038 |
| 161 | Gut microbiota abundance (genus Senegalimassilia id.11160) | 5 | MR Egger Weighted median Inverse variance weighted | 0.550981075 | 0.43094417 | 0.290998263 | 0.078693736 | 0.133346222 | 0.555092791 | 0.127453794 | 0.111906023 | 0.254729865 | 0.078605785 | 3 | 0.994274912 | 1.114320552 | 4 | 0.891993486 | -0.039589169 | 0.0389006 | 0.383747612 |
| 162 | Gut microbiota abundance (genus Slackia id.825) | 6 | MR Egger Weighted median Inverse variance weighted | 0.427252598 | 0.602899817 | 0.517649769 | -0.147632734 | 0.115260388 | 0.200241889 | -0.081365807 | 0.092716105 | 0.380171643 | 2.03078411 | 4 | 0.730096684 | 2.759717827 | 5 | 0.7369708 | -0.051203655 | 0.059973161 | 0.441342762 |
| 163 | Gut microbiota abundance (genus Streptococcus id.1853) | 14 | MR Egger Weighted median Inverse variance weighted | 0.020214663 | 0.37727403 | 0.958150798 | 0.007316988 | 0.136125272 | 0.957132855 | -0.097660837 | 0.100278069 | 0.330106065 | 14.30936577 | 12 | 0.281390572 | 14.43532733 | 13 | 0.343915753 | -0.009223141 | 0.028377844 | 0.750768324 |
| 164 | Gut microbiota abundance (genus Subdoligranulum id.2070) | 11 | MR Egger Weighted median Inverse variance weighted | 0.276502504 | 0.307453548 | 0.391902864 | -0.136500673 | 0.147526367 | 0.354829176 | -0.097913373 | 0.122458283 | 0.423962797 | 12.46408497 | 9 | 0.188392209 | 14.87388035 | 10 | 0.136728054 | -0.029559317 | 0.022408524 | 0.219705673 |
| 165 | Gut microbiota abundance (genus Sutterella id.2896) | 12 | MR Egger Weighted median Inverse variance weighted | -0.174119665 | 0.44434577 | 0.703385372 | -0.075324485 | 0.126203235 | 0.550607088 | -0.029733647 | 0.098226088 | 0.762113759 | 12.61331536 | 10 | 0.246102257 | 12.75397225 | 11 | 0.309709391 | 0.00992212 | 0.029712458 | 0.745323552 |
| 166 | Gut microbiota abundance (genus Terrisporobacter id.11348) | 5 | MR Egger Weighted median Inverse variance weighted | 0.089799456 | 0.51388372 | 0.872406618 | 0.077518945 | 0.146543429 | 0.596817419 | 0.233023352 | 0.149061571 | 0.117989307 | 7.721007191 | 3 | 0.052143653 | 7.945387712 | 4 | 0.093599322 | 0.014992093 | 0.050774546 | 0.787045203 |
| 167 | Gut microbiota abundance (genus Turicibacter id.2162) | 10 | MR Egger Weighted median Inverse variance weighted | -0.188870713 | 0.403363082 | 0.652105479 | 0.028036182 | 0.117955941 | 0.812126578 | 0.027213484 | 0.090174481 | 0.762814462 | 10.88208118 | 8 | 0.208470584 | 11.29480802 | 9 | 0.256041387 | 0.02331036 | 0.042318339 | 0.596790324 |
| 168 | Gut microbiota abundance (genus Tyzzerella3 id.11335) | 13 | MR Egger Weighted median Inverse variance weighted | -0.262993147 | 0.36414835 | 0.485233359 | 0.022582193 | 0.079085697 | 0.77522985 | 0.004504328 | 0.062318165 | 0.942379449 | 13.60045935 | 11 | 0.255895031 | 14.28856599 | 12 | 0.282661848 | 0.038394449 | 0.051466071 | 0.471300852 |
| 169 | Gut microbiota abundance (unknown genus id.1000000073) | 15 | MR Egger Weighted median Inverse variance weighted | 0.033256081 | 0.193012322 | 0.865854897 | 0.115259668 | 0.094036721 | 0.220316155 | 0.112954126 | 0.070585049 | 0.109541872 | 4.772567652 | 13 | 0.979864331 | 4.969390759 | 14 | 0.986234126 | 0.007797398 | 0.01757566 | 0.664591687 |
| 170 | Gut microbiota abundance (unknown genus id.1000001215) | 9 | MR Egger Weighted median Inverse variance weighted | -0.280060621 | 0.242574508 | 0.286168786 | 0.014247317 | 0.10263602 | 0.889597131 | -0.061248514 | 0.080803381 | 0.448454309 | 9.097365919 | 7 | 0.245740986 | 10.28839264 | 8 | 0.245365145 | 0.026424709 | 0.027603126 | 0.370298089 |
| 171 | Gut microbiota abundance (unknown genus id.1000005472) | 13 | MR Egger Weighted median Inverse variance weighted | -0.13646416 | 0.243128883 | 0.585857221 | -0.06649328 | 0.101456033 | 0.512216624 | -0.026797878 | 0.077043056 | 0.727967939 | 9.11798007 | 11 | 0.611003095 | 9.344147678 | 12 | 0.673288414 | 0.009440737 | 0.01985138 | 0.643684175 |
| 172 | Gut microbiota abundance (unknown genus id.1000005479) | 8 | MR Egger Weighted median Inverse variance weighted | -0.119506744 | 0.330761932 | 0.730251366 | 0.125018136 | 0.105117363 | 0.23431394 | 0.15827345 | 0.082408451 | 0.054782492 | 1.811478532 | 6 | 0.936194725 | 2.563453044 | 7 | 0.92224368 | 0.028159342 | 0.03247289 | 0.419175943 |
| 173 | Gut microbiota abundance (unknown genus id.1000006162) | 13 | MR Egger | -0.138695705 | 0.228815798 | 0.556719493 | -0.161834963 | 0.070377744 | 0.021475483 | -0.125014876 | 0.053728312 | 0.019976072 | 10.31062748 | 11 | 0.502681441 | 10.31441089 | 12 | 0.588398585 | 0.001620207 | 0.026340789 | 0.952056881 |
| 174 | Gut microbiota abundance (unknown genus id.1868) | 12 | MR Egger Weighted median Inverse variance weighted | -0.281793472 | 0.306331321 | 0.379281884 | -0.069772087 | 0.107485758 | 0.516255214 | -0.008060486 | 0.083924422 | 0.923485166 | 11.43306271 | 10 | 0.324787938 | 12.42107 | 11 | 0.332838783 | 0.025311018 | 0.027227692 | 0.374470931 |
| 175 | Gut microbiota abundance (unknown genus id.2001) | 10 | MR Egger Weighted median Inverse variance weighted | -0.00741568 | 0.284621702 | 0.979852055 | 0.031262943 | 0.114325351 | 0.784502778 | -0.114984496 | 0.097260244 | 0.237112338 | 12.88717997 | 8 | 0.115790797 | 13.15126973 | 9 | 0.155880084 | -0.011927579 | 0.02945849 | 0.696165625 |
| 176 | Gut microbiota abundance (unknown genus id.2041) | 12 | MR Egger Weighted median Inverse variance weighted | -0.117177268 | 0.233471439 | 0.626609373 | -0.117649811 | 0.097438508 | 0.227268035 | -0.11988169 | 0.071820382 | 0.095080208 | 6.592165903 | 10 | 0.763303969 | 6.592314106 | 11 | 0.831073088 | -0.000264493 | 0.021726378 | 0.99052637 |
| 177 | Gut microbiota abundance (unknown genus id.2071) | 15 | MR Egger Weighted median Inverse variance weighted | 0.082187254 | 0.406271157 | 0.842816405 | -0.048723497 | 0.107946963 | 0.651726394 | 0.004163635 | 0.08063578 | 0.958819466 | 10.11240107 | 13 | 0.684718113 | 10.15079607 | 14 | 0.751077522 | -0.006297405 | 0.032138405 | 0.847681832 |
| 178 | Gut microbiota abundance (unknown genus id.2755) | 13 | MR Egger Weighted median Inverse variance weighted | 0.288152562 | 0.244501413 | 0.263450402 | 0.016587992 | 0.087390689 | 0.849454742 | -0.010321062 | 0.065571593 | 0.87492849 | 6.589805926 | 11 | 0.831263154 | 8.195508983 | 12 | 0.769671427 | -0.030534097 | 0.024096417 | 0.231267084 |
| 179 | Gut microbiota abundance (unknown genus id.826) | 14 | MR Egger Weighted median Inverse variance weighted | 0.135888398 | 0.230434959 | 0.56632719 | 0.049203021 | 0.107428083 | 0.64694603 | 0.065725979 | 0.078809634 | 0.404289962 | 8.768049705 | 12 | 0.722604007 | 8.873036637 | 13 | 0.782463461 | -0.006698502 | 0.020673314 | 0.75150306 |
| 180 | Gut microbiota abundance (unknown genus id.959) | 11 | MR Egger Weighted median Inverse variance weighted | 0.048144189 | 0.296440627 | 0.874572827 | -0.092367241 | 0.072170187 | 0.200596792 | -0.041285313 | 0.057792605 | 0.474998312 | 2.961904452 | 9 | 0.965786165 | 3.056509537 | 10 | 0.980062723 | -0.010258764 | 0.033353223 | 0.7654011 |
| 181 | Gut microbiota abundance (genus Veillonella id.2198) | 6 | MR Egger Weighted median Inverse variance weighted | 1.013139114 | 0.8784646 | 0.313009627 | -0.09736497 | 0.137021914 | 0.477344822 | -0.018440107 | 0.110619416 | 0.867607169 | 2.315041327 | 4 | 0.6780319 | 3.7162355 | 5 | 0.590949742 | -0.078965405 | 0.066709503 | 0.302062917 |
| 182 | Gut microbiota abundance (genus Victivallis id.2256) | 10 | MR Egger Weighted median Inverse variance weighted | 0.140696938 | 0.403361722 | 0.736233743 | 0.064753439 | 0.068923367 | 0.347474571 | 0.038630008 | 0.052891166 | 0.465165354 | 3.766155949 | 8 | 0.877581331 | 3.831305727 | 9 | 0.922160458 | -0.013443546 | 0.052669279 | 0.804973795 |
| 183 | Gut microbiota abundance (order Actinomycetales id.420) | 5 | MR Egger Weighted median Inverse variance weighted | -0.122247269 | 0.231514128 | 0.634041648 | -0.100591403 | 0.123681228 | 0.416039311 | -0.101860262 | 0.101892814 | 0.317465139 | 0.675561071 | 3 | 0.878936615 | 0.685178431 | 4 | 0.953144066 | 0.002434543 | 0.02482501 | 0.928063228 |
| 184 | Gut microbiota abundance (order Bacillales id.1674) | 9 | MR Egger Weighted median Inverse variance weighted | 0.117986694 | 0.233865349 | 0.629395081 | -0.102007354 | 0.075025973 | 0.173947921 | -0.066956511 | 0.053104412 | 0.207364166 | 7.671913502 | 7 | 0.362410995 | 8.39632914 | 8 | 0.395743317 | -0.028602532 | 0.035181423 | 0.44298648 |
| 185 | Gut microbiota abundance (order Bacteroidales id.913) | 14 | MR Egger Weighted median Inverse variance weighted | 0.208130093 | 0.188290682 | 0.290670555 | 0.09062799 | 0.118539685 | 0.444547219 | 0.130107476 | 0.092264256 | 0.158492085 | 6.972701443 | 12 | 0.85941227 | 7.198661847 | 13 | 0.891625359 | -0.007055363 | 0.014842368 | 0.643070982 |
| 186 | Gut microbiota abundance (order Bifidobacteriales id.432) | 12 | MR Egger Weighted median Inverse variance weighted | 0.080693659 | 0.28126946 | 0.780052309 | 0.043636206 | 0.118492863 | 0.712679225 | 0.106242152 | 0.085546176 | 0.214263296 | 9.528348766 | 10 | 0.482799313 | 9.537440364 | 11 | 0.572410269 | 0.001978483 | 0.02074972 | 0.925920593 |
| 187 | Gut microbiota abundance (order Burkholderiales id.2874) | 10 | MR Egger Weighted median Inverse variance weighted | 0.128189346 | 0.346969631 | 0.721377202 | 0.028697933 | 0.144614305 | 0.8426972 | -0.005663096 | 0.109890812 | 0.958900135 | 5.198693334 | 8 | 0.736143784 | 5.364108866 | 9 | 0.80148352 | -0.009844267 | 0.024204452 | 0.694882326 |
| 188 | Gut microbiota abundance (order Clostridiales id.1863) | 13 | MR Egger Weighted median Inverse variance weighted | -0.051406726 | 0.264429535 | 0.84940168 | 0.129207167 | 0.130521526 | 0.32220836 | 0.059892958 | 0.096535213 | 0.534977341 | 8.256287471 | 11 | 0.69017864 | 8.460690379 | 12 | 0.748172903 | 0.008341388 | 0.018449934 | 0.659975481 |
| 189 | Gut microbiota abundance (order Coriobacteriales id.810) | 13 | MR Egger Weighted median Inverse variance weighted | 0.421998839 | 0.430067914 | 0.347564603 | 0.146630021 | 0.135657833 | 0.279749897 | 0.081714229 | 0.10137427 | 0.420205494 | 7.412472182 | 11 | 0.764765042 | 8.075355795 | 12 | 0.779214193 | -0.025138742 | 0.030876274 | 0.432815986 |
| 190 | Gut microbiota abundance (order Desulfovibrionales id.3156) | 12 | MR Egger Weighted median Inverse variance weighted | 0.451065961 | 0.253931905 | 0.106057892 | 0.035899379 | 0.13736692 | 0.793830837 | 0.067999252 | 0.108901064 | 0.532356318 | 12.325322 | 10 | 0.263873736 | 15.66134505 | 11 | 0.154176217 | -0.030711862 | 0.018667698 | 0.130956001 |
| 191 | Gut microbiota abundance (order Enterobacteriales id.3468) | 7 | MR Egger Weighted median Inverse variance weighted | 1.654847094 | 1.18155471 | 0.220244203 | -0.098773863 | 0.198042943 | 0.617955709 | 0.0116806 | 0.213373543 | 0.956343616 | 12.14477239 | 5 | 0.032857019 | 16.97765164 | 6 | 0.00936573 | -0.121473415 | 0.086116814 | 0.21744879 |
| 192 | Gut microbiota abundance (order Erysipelotrichales id.2148) | 13 | MR Egger Weighted median Inverse variance weighted | -0.860262742 | 0.458965255 | 0.087668191 | -0.013684954 | 0.139274059 | 0.921726496 | 0.023904398 | 0.105866523 | 0.821358906 | 4.664696412 | 11 | 0.946312113 | 8.584402105 | 12 | 0.737958051 | 0.055133712 | 0.027847775 | 0.073291265 |
| 193 | Gut microbiota abundance (order Gastranaerophilales id.1591) | 9 | MR Egger Weighted median Inverse variance weighted | -0.280060621 | 0.242574508 | 0.286168786 | 0.014247317 | 0.103644355 | 0.890664436 | -0.061248514 | 0.080803381 | 0.448454309 | 9.097365919 | 7 | 0.245740986 | 10.28839264 | 8 | 0.245365145 | 0.026424709 | 0.027603126 | 0.370298089 |
| 194 | Gut microbiota abundance (order Lactobacillales id.1800) | 15 | MR Egger Weighted median Inverse variance weighted | -0.176988836 | 0.255537766 | 0.500738257 | 0.00857633 | 0.13458353 | 0.949189228 | -0.069438254 | 0.096110566 | 0.469997549 | 15.2563808 | 13 | 0.291622068 | 15.50093363 | 14 | 0.344790999 | 0.008513682 | 0.018650272 | 0.655570275 |
| 195 | Gut microbiota abundance (order Methanobacteriales id.120) | 10 | MR Egger Weighted median Inverse variance weighted | -0.127169636 | 0.227713416 | 0.591810856 | -0.002712821 | 0.07208479 | 0.969979694 | 0.008005883 | 0.055482231 | 0.885266462 | 5.066778764 | 8 | 0.750415469 | 5.441404605 | 9 | 0.794258718 | 0.022495322 | 0.036753046 | 0.557484489 |
| 196 | Gut microbiota abundance (order Mollicutes RF9 id.11579) | 13 | MR Egger Weighted median Inverse variance weighted | -0.13646416 | 0.243128883 | 0.585857221 | -0.06649328 | 0.102225295 | 0.515396304 | -0.026797878 | 0.077043056 | 0.727967939 | 9.11798007 | 11 | 0.611003095 | 9.344147678 | 12 | 0.673288414 | 0.009440737 | 0.01985138 | 0.643684175 |
| 197 | Gut microbiota abundance (order NB1n id.3953) | 13 | MR Egger | -0.138695705 | 0.228815798 | 0.556719493 | -0.161834963 | 0.07021694 | 0.021178607 | -0.125014876 | 0.053728312 | 0.019976072 | 10.31062748 | 11 | 0.502681441 | 10.31441089 | 12 | 0.588398585 | 0.001620207 | 0.026340789 | 0.952056881 |
| 198 | Gut microbiota abundance (order Pasteurellales id.3688) | 14 | MR Egger Weighted median Inverse variance weighted | 0.005291576 | 0.137780148 | 0.969995567 | -0.012749315 | 0.086132912 | 0.882327707 | -0.015997871 | 0.063435923 | 0.800894633 | 7.014718182 | 12 | 0.856639381 | 7.045016523 | 13 | 0.899816784 | -0.002827712 | 0.016245223 | 0.864716999 |
| 199 | Gut microbiota abundance (order Rhodospirillales id.2667) | 14 | MR Egger Weighted median Inverse variance weighted | 0.212643801 | 0.265114454 | 0.438100998 | 0.028800682 | 0.08267568 | 0.727571817 | -0.021334591 | 0.065514482 | 0.744691487 | 4.913277498 | 12 | 0.960812924 | 5.742842019 | 13 | 0.955013246 | -0.023567445 | 0.025875421 | 0.380330522 |
| 200 | Gut microbiota abundance (order Selenomonadales id.2165) | 12 | MR Egger Weighted median Inverse variance weighted | 0.355217054 | 0.365118963 | 0.353557502 | 0.120393326 | 0.141054756 | 0.393369936 | 0.111583722 | 0.105775231 | 0.291464875 | 9.398166552 | 10 | 0.494778323 | 9.884209135 | 11 | 0.540831802 | -0.016081593 | 0.023067042 | 0.501583663 |
| 201 | Gut microbiota abundance (order Verrucomicrobiales id.4030) | 11 | MR Egger Weighted median Inverse variance weighted | -0.284588142 | 0.294773938 | 0.359545603 | 0.097834247 | 0.1205647 | 0.417097672 | 0.054441114 | 0.088140546 | 0.53679781 | 5.710304567 | 9 | 0.768525496 | 7.162991578 | 10 | 0.709973171 | 0.027944777 | 0.023185401 | 0.258830128 |
| 202 | Gut microbiota abundance (order Victivallales id.2254) | 8 | MR Egger Weighted median Inverse variance weighted | 0.187773836 | 0.289649692 | 0.540804736 | -0.081767544 | 0.086969949 | 0.347124431 | -0.024107539 | 0.079248792 | 0.760974983 | 8.818278109 | 6 | 0.184058862 | 9.672763288 | 7 | 0.207888397 | -0.031507442 | 0.041321602 | 0.474664226 |
| 203 | Gut microbiota abundance (phylum Actinobacteria id.400) | 15 | MR Egger Weighted median Inverse variance weighted | 0.505163612 | 0.393741081 | 0.221899673 | 0.010159988 | 0.12612001 | 0.935793393 | -0.01315872 | 0.092650751 | 0.887060284 | 7.360829061 | 13 | 0.882611874 | 9.195325026 | 14 | 0.81833812 | -0.032230569 | 0.023796309 | 0.1986716 |
| 204 | Gut microbiota abundance (phylum Bacteroidetes id.905) | 11 | MR Egger Weighted median Inverse variance weighted | 0.171251171 | 0.308150497 | 0.591934136 | 0.033309151 | 0.149421594 | 0.823597303 | 0.039209137 | 0.129868037 | 0.76271669 | 13.94030054 | 9 | 0.124464757 | 14.29249178 | 10 | 0.160063317 | -0.010432312 | 0.021877925 | 0.644839703 |
| 205 | Gut microbiota abundance (phylum Cyanobacteria id.1500) | 8 | MR Egger Weighted median Inverse variance weighted | -0.043449742 | 0.356077967 | 0.906865033 | -0.087655464 | 0.11028604 | 0.426729222 | -0.016367056 | 0.092603917 | 0.859710586 | 9.094660019 | 6 | 0.168324031 | 9.104178904 | 7 | 0.245262747 | 0.003284745 | 0.041450165 | 0.939414151 |
| 206 | Gut microbiota abundance (phylum Euryarchaeota id.55) | 12 | MR Egger Weighted median Inverse variance weighted | -0.098168736 | 0.2258866 | 0.67308613 | -0.050972045 | 0.070812162 | 0.471635531 | 0.007087991 | 0.049289584 | 0.885656023 | 10.70735619 | 10 | 0.380767511 | 10.95233374 | 11 | 0.447265231 | 0.015224414 | 0.031828675 | 0.642704965 |
| 207 | Gut microbiota abundance (phylum Firmicutes id.1672) | 15 | MR Egger Weighted median Inverse variance weighted | 0.10721425 | 0.229336178 | 0.647883177 | 0.005173612 | 0.120212641 | 0.965671903 | -0.013466522 | 0.095566073 | 0.88793851 | 16.59008087 | 13 | 0.218726973 | 17.02221471 | 14 | 0.254995967 | -0.010324163 | 0.017741825 | 0.570580639 |
| 208 | Gut microbiota abundance (phylum Lentisphaerae id.2238) | 9 | MR Egger Weighted median Inverse variance weighted | 0.253640829 | 0.320597992 | 0.454823186 | -0.062081317 | 0.087461898 | 0.47782206 | 0.019692485 | 0.082181645 | 0.81062373 | 12.53120225 | 7 | 0.084388415 | 13.55557988 | 8 | 0.094110108 | -0.035125174 | 0.046433967 | 0.474061209 |
| 209 | Gut microbiota abundance (phylum Proteobacteria id.2375) | 12 | MR Egger Weighted median Inverse variance weighted | -0.112039159 | 0.2814406 | 0.69892891 | 0.160714658 | 0.13163934 | 0.222134852 | 0.135966321 | 0.101038118 | 0.178401442 | 3.065536385 | 10 | 0.979839392 | 3.956936671 | 11 | 0.971159814 | 0.017508987 | 0.018544906 | 0.36735006 |
| 210 | Gut microbiota abundance (phylum Tenericutes id.3919) | 12 | MR Egger Weighted median Inverse variance weighted | -0.035500494 | 0.396350725 | 0.93039874 | -0.062783214 | 0.118351516 | 0.595778574 | -0.041406464 | 0.110426712 | 0.707684399 | 19.15653743 | 10 | 0.038318881 | 19.15700248 | 11 | 0.058332832 | -0.000538391 | 0.034554625 | 0.98787524 |
| 211 | Gut microbiota abundance (phylum Verrucomicrobia id.3982) | 12 | MR Egger Weighted median Inverse variance weighted | -0.008490422 | 0.218753023 | 0.96980355 | -0.039580304 | 0.116720998 | 0.734533021 | -0.043124461 | 0.083622528 | 0.606061252 | 8.593748642 | 10 | 0.571042317 | 8.623105275 | 11 | 0.656636448 | -0.003306284 | 0.019296878 | 0.867375617 |
